# *Tet2* deficiency–induced expansion of monocyte-derived macrophages promotes liver fibrosis

**DOI:** 10.1084/jem.20251114

**Published:** 2025-12-26

**Authors:** Jiuxing Feng, Baitong Wu, Yu Li, Pengli Sun, Qian Liu, Qianxue Xiao, Jia-Bin Cai, Yimin Zheng, Haonan Chen, Yichi Xu, Yixin Liu, Guo-Ming Shi, Li Tan, Yujiang Geno Shi

**Affiliations:** 1 The Shanghai Key Laboratory of Medical Epigenetics, Liver Cancer Institute, Institutes of Biomedical Sciences and Zhongshan Hospital, Fudan University, Shanghai, P.R. China; 2 East Hospital, Stem Cell Research Center, School of Medicine, Tongji University, Shanghai, P.R. China; 3 Center for Medical Research and Innovation, Shanghai Pudong Hospital, Fudan University Pudong Medical Centre and Shanghai Key Laboratory of Medical Epigenetics, Fudan University, Shanghai, PR. China; 4 Department of Biomedical Engineering, Cornell University, Ithaca, NY, USA

## Abstract

Clonal hematopoiesis driven by *Tet2* deficiency in myeloid cells (*Tet*^ΔMye^) is prevalent in elderly individuals; however, the role of *Tet2*^ΔMye^ in liver fibrosis pathogenesis remains elusive. In this study, we demonstrated that *Tet2*-deficient monocyte-derived macrophages (MDMs) promoted cellular expansion and elevated C–C motif chemokine ligand 2/8 (Ccl2/8) secretion by stabilizing their mRNAs through 5hmC-mediated alterations in RNA–protein interactions. These chemokines engaged with the upregulated C–C motif chemokine receptor (Ccr2/3) on *Tet2*^−/−^ monocytes, forming a positive feedback loop that amplified pro-inflammatory MDMs (pMDMs) accumulation in liver. *Tet2*^−/−^ pMDMs activated hepatic stellate cells through IL-6, driving extracellular matrix deposition and fibrotic progression. Pharmacological inhibition of Ccl2/Ccl8 with Bindarit attenuated MDMs accumulation and liver fibrosis, whereas combined therapy with Bindarit and IL-6 neutralization synergistically suppressed liver fibrosis in *Tet2*^ΔMye^ mice and aged chimeric models recapitulating *Tet2*^ΔMye^-related myeloid hematopoiesis. These findings present the mechanism that *Tet2*^ΔMye^ aggravates liver fibrosis and highlight MDMs depletion plus IL-6 neutralization as a promising therapy for liver fibrosis in patients with *Tet2*^ΔMye^-related myeloid hematopoiesis.

## Teaser

Tet2^ΔMye^ exacerbates liver fibrosis through stabilizing Ccl2/Ccl8 mRNAs in pMDMs, creating a chemokine-driven inflammatory loop. Targeting this axis with Bindarit and IL-6 blockade synergistically reduces fibrosis associated with Tet2^ΔMye^-related myeloid hematopoiesis.

## Graphical Abstract

**Figure:**
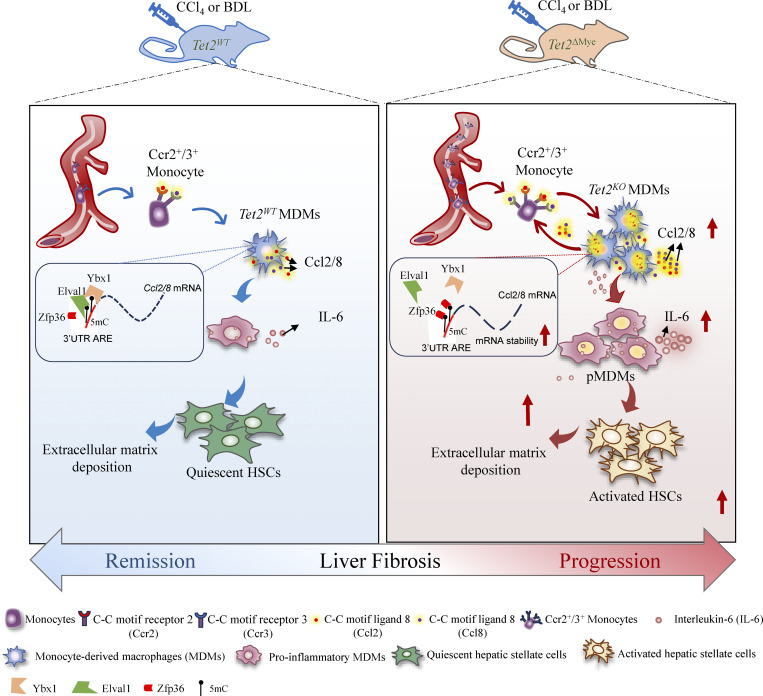

## Introduction

Liver fibrosis is a pathological response to chronic liver injury, characterized by excessive deposition of extracellular matrix components, which disrupts liver architecture and function (Altamirano-Barrera et al., 2017). Globally, over half a million individuals progress from liver fibrosis to cirrhosis and ultimately to hepatocellular carcinoma annually (Doycheva et al., 2017; Friedman and Pinzani, 2022). Liver fibrosis develops as an immunogenic response to chronic injury, where monocyte and macrophage-driven inflammation activate collagen-producing cells—particularly hepatic stellate cells (HSCs), portal fibroblasts, and myofibroblasts—leading to pathological extracellular matrix accumulation (Asano et al., 2015; Heymann et al., 2009). During chronic liver injury, Ly6c^high^ monocytes infiltrate the liver and differentiate into pro-inflammatory monocyte-derived macrophages (pMDMs), whereas Ly6c^low^ monocytes contribute to fibrotic “repair” macrophages (Baeck et al., 2014). Although targeting monocytes and MDMs could attenuate methionine-choline–deficient diet-induced liver fibrosis and nonalcoholic steatohepatitis progression in mouse models (Baeck et al., 2014; Ratziu et al., 2020; Sano et al., 2000; Chalasani et al., 2020), effective clinical therapies remain limited (Anstee et al., 2020; Ratziu et al., 2020; Sano et al., 2000).

Emerging evidence highlights the anti-inflammatory role of 10–11 translocation 2 (*Tet2*) in monocytes and macrophages (Fuster et al., 2017; Zhang et al., 2015; Cull et al., 2017; Lv et al., 2018). However, *TET2* mutations can lead to clonal hematopoiesis of indeterminate potential (CHIP), a condition characterized by the skewed expansion of *TET2*-mutated hematopoietic stem cells and their progeny (Asada and Kitamura, 2021). *Tet2*-deficient hematopoietic stem cells preferentially differentiate into pro-inflammatory myeloid cells, which exhibit enhanced migration to injured liver and tissues (Marchetti et al., 2024; Wong et al., 2023). Notably, *TET2* mutations in myeloid cells are detected in ∼10% of individuals over 65 years old, making CHIP significantly more prevalent in the elderly (Ferrone et al., 2020; Jaiswal and Ebert, 2019; Genovese et al., 2014).

The prevalence of liver fibrosis also increases dramatically with age, affecting over 25% of individuals aged 65 and older, even in the absence of overt liver disease (Udomsinprasert et al., 2021). This age-related susceptibility is closely associated with chronic low-grade inflammation and immune dysregulation (Franceschi et al., 2018). Previous studies have demonstrated that *TET2* mutation–induced CHIP is positively correlated with both aging and the progression of advanced liver fibrosis (Marchetti et al., 2024). Recent research has further shown that age-dependent *TET2* mutations promote CHIP and enhance susceptibility to chronic liver diseases by upregulating IL-6 and activating the NLRP3 inflammasome (Wong et al., 2023), indicating a potential mechanistic link between *TET2* dysfunction and age-associated liver fibrosis progression.

Although previous studies have focused on *Tet2* mutations in hematopoietic stem cells, this study emphasizes the role of *Tet2* mutations specifically in myeloid cells (*Tet2*^ΔMye^). Because of the pivotal role of myeloid cells during liver fibrosis and the age-associated accumulation of *Tet2* mutations in myeloid cells, a critical question arises: Does *Tet2*^ΔMye^-driven myeloid cell skewing exacerbate fibrosis progression during chronic liver injury? This question gains heightened significance in the context of aging, where the concurrent rise in *Tet2* mutation prevalence and liver fibrosis incidence underscores the urgent need to elucidate these mechanisms for potential therapeutic interventions.

In this study, we addressed this critical question by investigating the novel mechanism of *Tet2*^ΔMye^ in liver fibrosis progression. We demonstrated that *Tet2*^ΔMye^ promotes fibrosis through two interconnected pathways: a C–C motif chemokine ligand 2 (Ccl2)- and C–C motif chemokine ligand 8 (Ccl8)-mediated positive feedback loop, facilitated by their enhanced binding to upregulated C–C motif chemokine receptor 2/3 (Ccr2/3) on *Tet2*^−/−^ monocytes, which drives the expansion of pMDMs in liver; and (2) the activation of HSCs mediated by IL-6 secreted from pMDMs. Importantly, these findings were validated in both aging and young liver fibrosis models, highlighting the universal role of *Tet2*^ΔMye^ in fibrosis progression, while highlighting its heightened impact in the context of aging. These findings provide novel insights into the pathogenesis of *Tet2*^ΔMye^-associated liver fibrosis and establish a strong rationale for developing therapies targeting MDM populations and *Il-6* signaling. The demonstrated efficacy of this approach in preclinical models supports its potential as a therapeutic strategy for age-related liver fibrosis in patients with *Tet2*^ΔMye^-related CHIP.

## Results

### Loss of *Tet2* in myeloid cells exacerbates liver fibrosis

**Figure S1.**
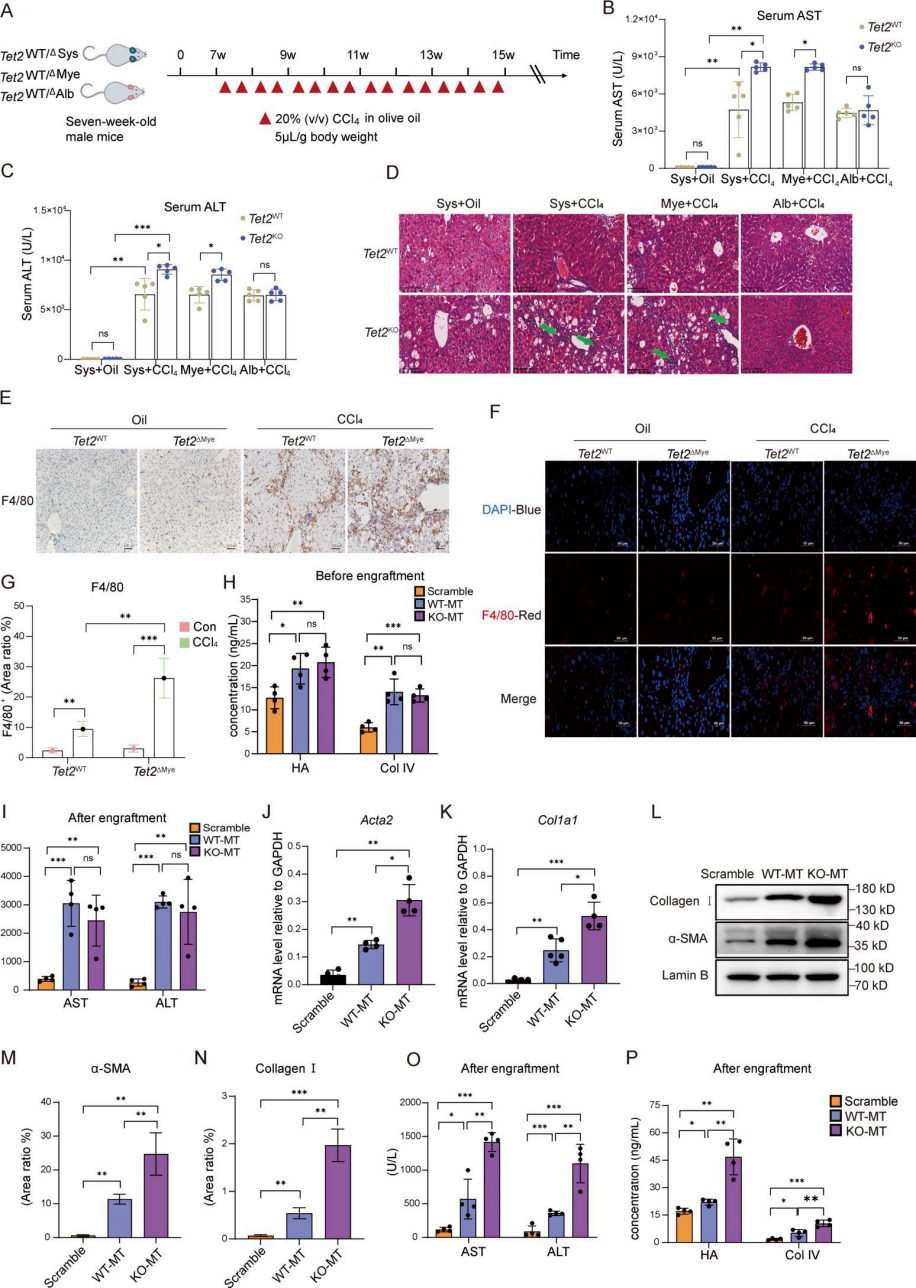
**Effect of *Tet2*** ^**ΔMye**^ **on hepatic fibrosis progression. (A)** Treatment schedule for the construction of a CCl_4_-induced mouse model in systemic *Tet2* KO (*Tet2*^ΔSys^) mice, hepatocyte-specific *Tet2* KO (*Tet2*^ΔAlb^) mice, and myeloid cell–specific *Tet2* KO (*Tet2*^ΔMye^) mice. 7-wk-old male littermates (*Tet2* WT or KO) were used to construct a liver fibrosis model. **(B and C)** Serum levels of AST (B) and ALT (C) in different types of *Tet2*-deficient mice with liver fibrosis (*n* = 5 for each group). **(D)** H&E staining of livers in different types of *Tet2*-deficient liver fibrosis mouse models. Green arrows indicate infiltrated immune cells in liver tissues. **(E)** IHC staining of F4/80 in liver tissues of *Tet2*^ΔMye^ mice treated with oil or CCl_4_ (*n* = 4 for each group). Scale bar, 100 μm. **(F and G)** IF staining (F) and statistical analysis (G) of F4/80 in liver tissues of *Tet2*^ΔMye^ mice treated with oil or CCl_4_ (*n* = 4 for each group). Blue: DAPI; red: anti-F4/80. **(H and I)** Serum levels of AST and ALT measured by serum analyzer in CD45.1 mice before (H) and after (I) transplantation of CD11b^+^*Tet2*^+/+^ or CD11b^+^*Tet2*^−/−^ myeloid cells (*n* = 4 for each group). **(J and K)** mRNA levels of *Acta2* (J) and *Col1a1* (K) in livers of scramble, WT-MT, and KO-MT mice (*n* = 4 for each group). **(L)** Expression levels of α-SMA and collagen I measured by western blot. **(M and N)** Statistical analysis of α-SMA (M) and collagen I (N)-positive area ratio for IHC staining in liver of scramble, WT-MT, and KO-MT mice (*n* = 4 for each group). **(O)** Serum levels of AST and ALT measured by serum analyzer in scramble, WT-MT, and KO-MT mice (*n* = 4 for each group). **(P)** Serum levels of HA and Col IV measured by ELISA in scramble, WT-MT, and KO-MT mice (*n* = 4 for each group). Data are representative of at least two independent experiments with similar results (B–O). All data are shown as mean ± SD and were analyzed by two-way ANOVA with Tukey’s multiple comparison test (B, C, and G) or one-way ANOVA with Tukey’s multiple comparison test (H–K and M–P). ***P < 0.001; **P < 0.01; *P < 0.05; P > 0.05 not significant (ns). Source data are available for this figure: SourceData FS1.

**Figure 1.**
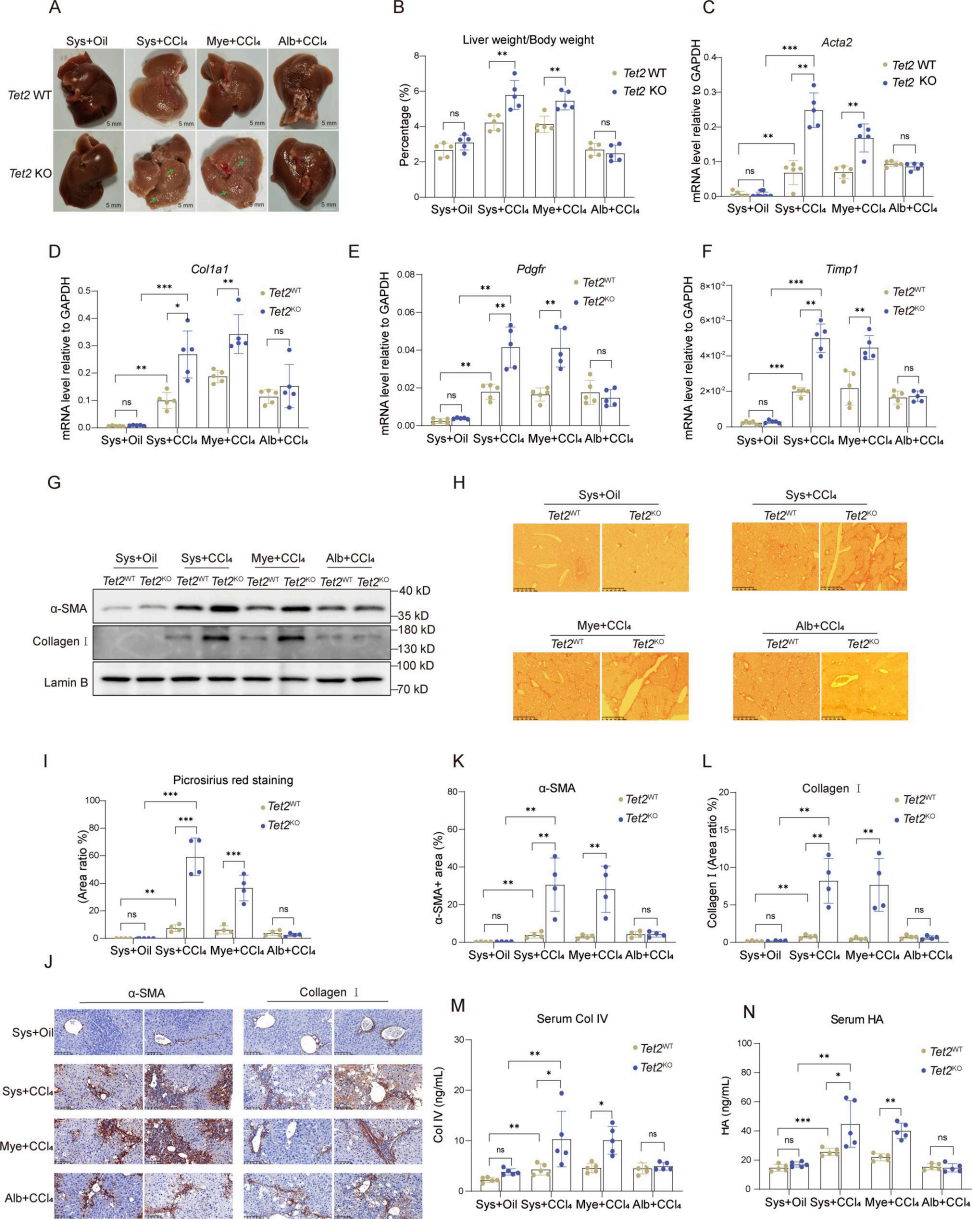
**Loss of *Tet2* in myeloid cells exacerbates liver fibrosis. (A)** Representative pictures of fibrotic liver from three types of *Tet2*-deficient mice treated with olive oil or CCl_4_. Sys, systemic *Tet2* KO; Mye: myeloid cell–specific *Tet2* KO; Alb: hepatocyte-specific *Tet2* KO. 7-wk-old *Tet2*-deficient and *Tet2*-WT littermates were used to establish liver fibrosis model (*n* = 5 for each group). Pathological liver fibrosis lesions are indicated by green arrows in the *Tet2*^ΔSys^ and *Tet2*^ΔMye^ groups. Scale bar, 5 mm. **(B)** Statistical analysis of liver/body weight ratios of each group as shown in Fig. 1 A (*n* = 5 for each group). **(C–F)** mRNA levels of *Acta2* (C), *Col1a1* (D), *Pdgfr* (E), and *Timp1* (F) in liver tissues of mice depicted in Fig. 1 A (*n* = 5 for each group). Gene expression levels were normalized to *Gapdh*. **(G)** Protein levels of collagen I and α-SMA in the liver tissues of different groups of mice were quantified by western blotting assay. Protein expression levels were normalized to Lamin B. **(H and I)** The Picrosirius red staining (scale bar, 125 μm) (H) and statistical analysis (I) of ratio of positive staining area (*n* = 4 for each group). Statistical analysis was performed using Image-Pro Plus 6.0. **(J–L)** IHC staining (scale bar, 100 μm) (J) and statistical analysis of α-SMA (K) and collagen I (L) of ratio of positive staining area of liver tissues to analyze collagen deposition and expression in different groups of mice. Statistical analysis was performed using Image-Pro Plus 6.0 (*n* = 4 for each group). **(M and N)** Serum levels of Col IV (M) and HA (N) in different types of *Tet2*-deficient mice with liver fibrosis (*n* = 5 for each group). Data are representative of at least two independent experiments with similar results (A–N). All data are shown as mean ± SD and were analyzed by two-way ANOVA with Tukey’s multiple comparison test (B–F, I, and K–N). ***P < 0.001; **P < 0.01; *P < 0.05; P > 0.05 not significant (ns). *Timp1*, TIMP metallopeptidase inhibitor 1; *Pdgfr*, platelet-derived growth factor receptor. Source data are available for this figure: SourceData F1.

To investigate the impact of *Tet2* deficiency on liver fibrosis progression under chronic injury, we established a carbon tetrachloride (CCl_4_)-induced liver fibrosis model in systemic *Tet2* KO (*Tet2*^ΔSys^) mice, hepatocyte-specific *Tet2* KO (*Tet2*^ΔAlb^) mice, and myeloid cell–specific *Tet2* KO (*Tet2*^ΔMye^) mice (Fig. S1 A). Gross morphological analysis revealed that *Tet2*^ΔSys^-CCl_4_ and *Tet2*^ΔMye^-CCl_4_ mice exhibited rounder and blunter hepatic edges with more uneven and granular liver surfaces compared with *Tet2*^ΔAlb^-CCl_4_ and WT littermate controls, indicating that *Tet2*^ΔSys^-CCl_4_ and *Tet2*^ΔMye^-CCl_4_ mice developed more severe fibrosis (Fig. 1 A). Quantitative assessments revealed that *Tet2*^ΔSys^-CCl_4_ and *Tet2*^ΔMye^-CCl_4_ mice exhibited significantly increased liver/body weight ratios (Fig. 1 B) and elevated mRNA levels of fibrosis markers, including *Acta2*, collagen type I alpha 1 chain (*Col1a1*), *Pdgfr*, and *Timp1* (Fig. 1, C–F). Compared with *Tet2*^WT^ littermates, *Tet2*^ΔSys^-CCl_4_ and *Tet2*^ΔMye^-CCl_4_ mice showed upregulated protein expression of alpha-smooth muscle actin (α-SMA) and collagen I in liver tissues (Fig. 1 G), along with increased collagens and α-SMA *in situ* deposition (Fig. 1, H–L). Serum analysis revealed higher levels of collagen type IV (Col IV) (Fig. 1 M) and hyaluronic acid (HA) (Fig. 1 N), as well as elevated alanine aminotransferase (ALT) and aspartate aminotransferase (AST) activities (Fig. S1, B and C), in *Tet2*^ΔSys^-CCl_4_ and *Tet2*^ΔMye^-CCl_4_ mice compared with littermate controls. Histological examination demonstrated substantial F4/80^+^ macrophage infiltration around intrahepatic vessels in *Tet2*^ΔMye^-CCl_4_ mice (Fig. S1, D–G). In contrast, *Tet2*^ΔAlb^ mice showed no significant differences in collagen I and α-SMA deposition, HA and Col IV levels, AST and ALT activities, and F4/80^+^ macrophage infiltration compared with littermate controls (Fig. 1, A–N; and Fig. S1, B–G), indicating that *Tet2*^ΔAlb^ does not markedly influence liver fibrosis progression. Collectively, these findings demonstrate that myeloid-specific, but not hepatocyte-specific, *Tet2* deletion exacerbates CCl_4_-induced liver fibrosis in mice, highlighting the cell type–specific role of *Tet2* in liver fibrogenesis.

### *Tet2* ^ΔMye^ promotes intrahepatic expansion of pMDMs in liver

**Figure 2.**
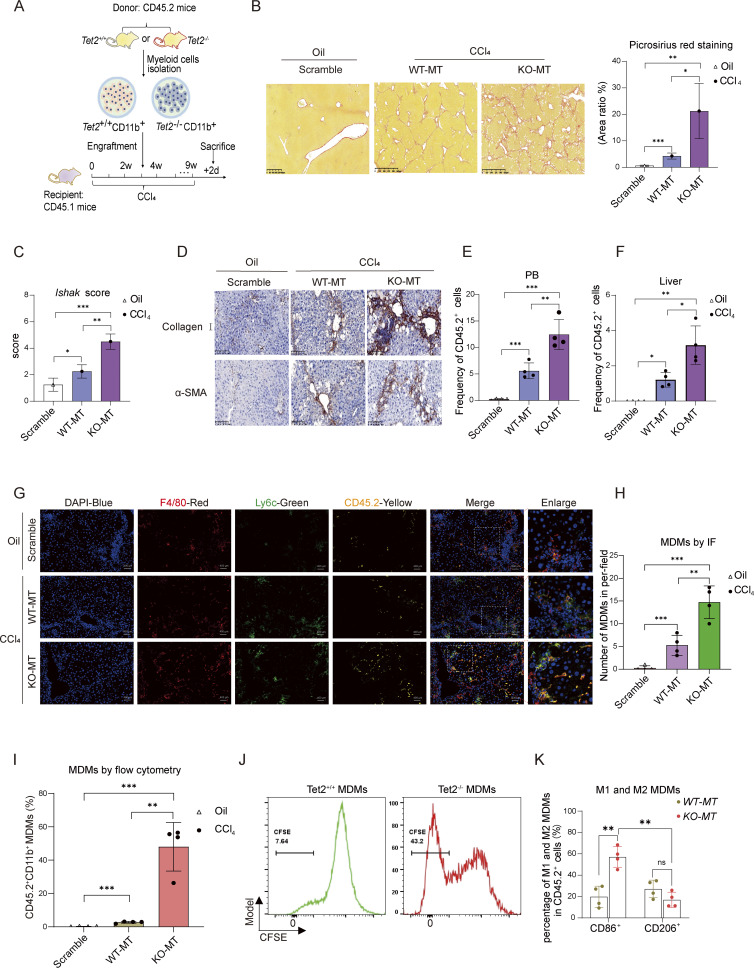
***Tet2*** ^**ΔMye**^ **promoted intrahepatic expansion of pMDMs in liver fibrosis model. (A)** Chimeric mice with liver fibrosis were constructed by transplantation of PBS (scramble), CD45.2^+^*Tet2*^+/+^CD11b^+^ myeloid cells (WT-MT), or CD45.2^+^*Tet2*^−/−^CD11b^+^ myeloid cells (KO-MT) into CD45.1 recipient mice, followed by repeated CCl_4_ treatment (*n* = 4 for each group). CD45.2^+^*Tet2*^+/+^ CD11b^+^ and CD45.2^+^*Tet2*^−/−^ CD11b^+^ cells were isolated from CD45.2 *Tet2*^+/+^ and *Tet2*^−/−^ littermates. **(B)** Picrosirius red staining (scale bar, 125 μm) and statistical analysis of liver fibrosis in scramble, WT-MT, and KO-MT mice (*n* = 4 for each group). **(C)** Ishak score evaluation based on Sirius red staining and H&E staining in livers of scramble, WT-MT, and KO-MT mice (*n* = 4 for each group). **(D)** Expression levels of collagen I and α-SMA were measured by IHC (scale bar, 100 μm). **(E and F)** Frequency of CD45.2^+^ cell populations in peripheral blood (E) and liver (F) of scramble, WT-MT, and KO-MT mice (*n* = 4 for each group). **(G and H)** IF (G) (scale bar, 50 μm) and statistical analysis (H) of CD45.2^+^ MDMs in livers of scramble, WT-MT, and KO-MT mice (*n* = 4 for each group). Yellow: anti-CD45.2, blue: DAPI, red: anti-F4/80, and green: anti-Ly6c (*n* = 4). **(I)** Statistical analysis of the frequency of CD45.2^+^ MDMs in livers of scramble, WT-MT, and KO-MT mice evaluated by flow cytometry (*n* = 4 for each group). **(J)** Expansion of *Tet2*^−/−^ pMDMs and *Tet2*^−/−^ pMDMs was quantified by CFSE staining *in vitro*. Representative histograms show differences in CFSE signals between the two groups (*n* = 3 for each group). **(K)** Frequency of CD86^+^ MDMs (M1) and CD206^+^ MDMs (M2) evaluated by flow cytometry in the liver of WT-MT and KO-MT mice (*n* = 4 for each group). Data are representative of at least two independent experiments with similar results (B–K). All data are shown as mean ± SD and were analyzed by one-way ANOVA with Tukey’s multiple comparison test (B, C, E, F, H, and I) or two-way ANOVA with Sidak’s multiple comparison test (K). ***P < 0.001; **P < 0.01; *P < 0.05; P > 0.05 not significant (ns).

To elucidate the mechanism through which *Tet2*-deficient myeloid cells exacerbate liver fibrosis, we established a competitive myeloid chimeric mouse model through transplantation (Fig. 2 A). Lethally irradiated CD45.1 WT recipient mice were intravenously transplanted with a mixture of 20% CD45.2^+^*Tet2*^−/−^ CD11b^+^ cells and 80% CD45.2^+^*Tet2*^+/+^CD11b^+^ cells (designated “KO-MT”). Control recipient mice received 20% CD45.2^+^*Tet2*^+/+^CD11b^+^ cells mixed with 80% CD45.2^+^*Tet2*^+/+^CD11b^+^ cells (designated “WT-MT”). Following transplantation, mice were administrated with CCl_4_ to induce liver fibrosis model. Prior to engraftment, serum levels of AST, ALT, HA, and Col IV were comparable across groups (Fig. S1, H and I). After another 3 wk of CCl_4_ treatment, KO-MT mice exhibited significantly elevated collagen deposition (Fig. 2 B) and higher Ishak fibrosis scores (Fig. 2 C) compared with WT-MT and scramble controls. Immunohistochemical (IHC) analysis revealed increased intrahepatic expression of α-SMA and collagen I in KO-MT mice (Fig. 2 D and Fig. S1, J–N), alongside elevated serum levels of AST, ALT (Fig. S1 O), HA, and Col IV (Fig. S1 P). These findings demonstrate that *Tet2*^−/−^ myeloid cell engraftment is sufficient to exacerbate CCl_4_-induced liver fibrosis.

**Figure S2.**
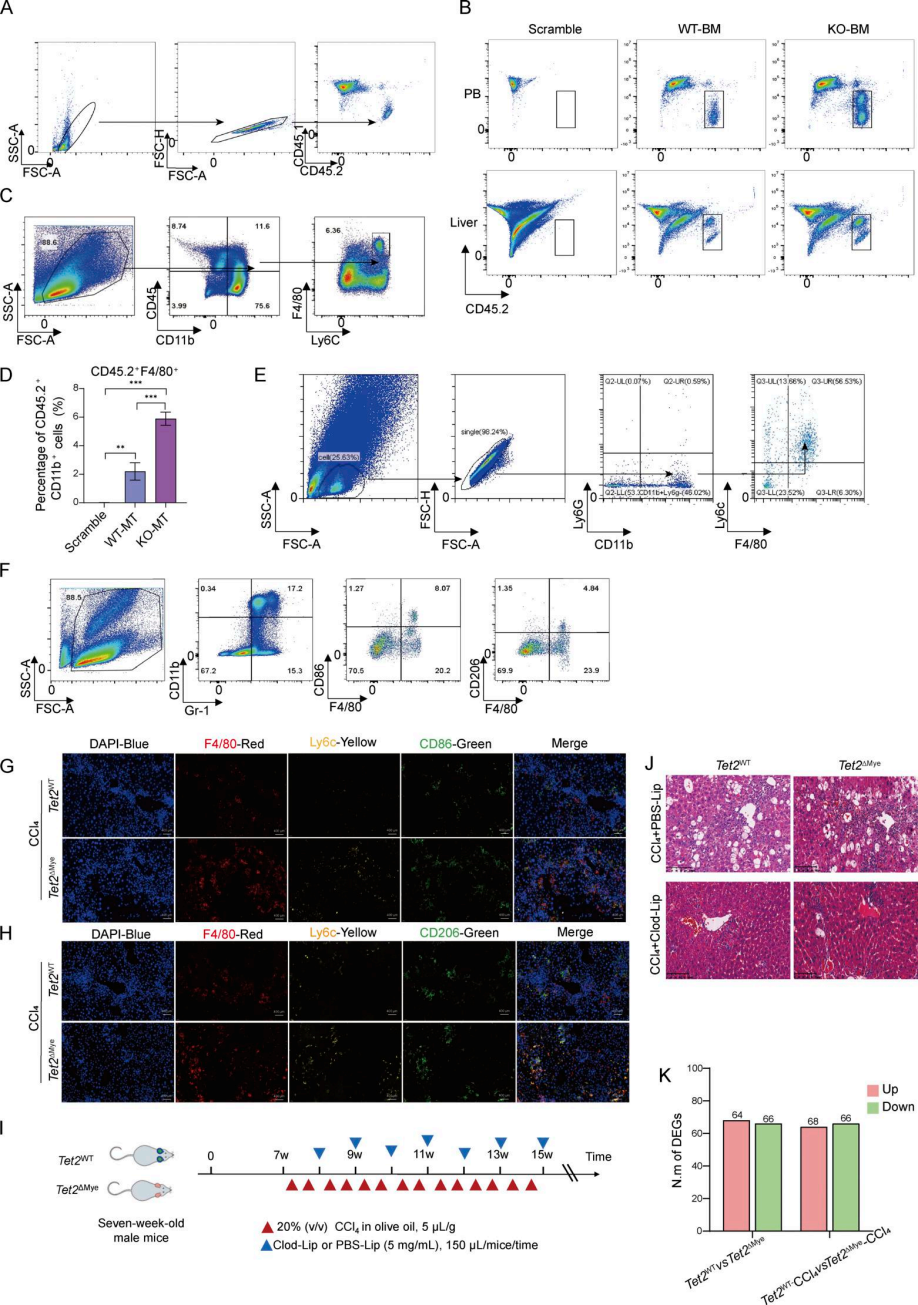
**Expansion advantage of transplanted *Tet2*** ^**−/−**^ **CD45.2** ^**+**^ **CD11b** ^**+**^ **cells and change of immune cells after Clod-Lip treatment in *Tet2*** ^**WT**^ **-CCl**_**4**_ **and *Tet2*** ^**ΔMye**^ **-CCl**_**4**_ **mice. (A)** Strategy for flow cytometric analysis of the proportion of CD45.2^+^ cells in the blood and liver of scramble, WT-MT, and KO-MT mice. **(B)** Representative graphs of CD45.2^+^ cells in peripheral blood and liver of scramble, WT-MT, and KO-MT mice. **(C)** Flow cytometry strategy and statistical analysis of CD45.2^+^ F4/80^+^ cell frequency in livers of scramble, WT-MT, and KO-MT mice (*n* = 4). **(D)** Flow cytometry strategy for MDMs frequency in liver tissue of scramble, WT-MT, and KO-MT mice. **(E and F)** Strategy for flow cytometry analysis of MDMs (F), (CD86^+^) M1, and (CD206^+^) M2 macrophages (G) in livers of PBS-Lip– or Clod-Lip–treated *Tet2*^WT^-CCl_4_ and *Tet2*^ΔMye^-CCl_4_ mice. M1 macrophages were defined as Gr1^+^CD11b^+^F4/80^+^CD86^+^; M2 macrophages were defined as Gr1^+^CD11b^+^ F4/80^+^CD206^+^ populations. **(G and H)** IF (scale bar, 400 μm) of M1 MDMs (CD86) (G) and M2 MDMs (CD206) (H) in livers of *Tet2*^WT^-CCl_4_ and *Tet2*^ΔMye^-CCl_4_ mice. Blue: DAPI, red: anti-F4/80, green: anti-CD86/CD163, and yellow: anti-Ly6c. **(I)** Treatment schedule of PBS-Lip or Clod-Lip in *Tet2*^WT^-CCl_4_ and *Tet2*^ΔMye^-CCl_4_ mice. **(J)** H&E staining of liver tissues treated with PBS-Lip or Clod-Lip in *Tet2*^WT^-CCl_4_ and *Tet2*^ΔMye^-CCl_4_ mice. **(K)** Statistical analysis of DEGs in the comparison of *Tet2*^WT^ versus *Tet2*^ΔMye^ mice and *Tet2*^WT^-CCl_4_ versus *Tet2*^ΔMye^-CCl_4_ mice. A twofold decrease or increase in gene expression was considered statistically significant between different groups (*Tet2*^WT^, *n* = 4; *Tet2*^ΔMye^, *n* = 4; *Tet2*^WT^-CCl_4_, *n* = 5; *Tet2*^ΔMye^-CCl_4_, *n* = 5). Data are representative of at least two independent experiments with similar results (B, D, G, and F). All data are shown as mean ± SD and were analyzed by one-way ANOVA with Tukey’s multiple comparison test (D). ***P < 0.001; **P < 0.01; *P < 0.05; P > 0.05 not significant (ns).

To assess the distribution and lineage transformation of engrafted CD45.2^+^ myeloid cells, flow cytometry was performed. KO-MT mice exhibited a higher frequency of CD45.2^+^ cells in both peripheral blood and liver in comparison with controls (Fig. 2, E and F; and Fig. S2, A and B), indicating enhanced expansion capacity of *Tet2*^−/−^ myeloid cells. Notably, KO-MT mice showed increased infiltration of CD45.2^+^F4/80^+^ macrophages, identified as Ly6c^high^ MDMs (Fig. 2, G–I; and Fig. S2, C–E). *In vitro* assays confirmed that *Tet2*^−/−^ MDMs exhibited greater proliferative capacity than *Tet2*^−/−^ MDMs (Fig. 2 J). Furthermore, KO-MT mice displayed a higher proportion of CD86^+^ pMDMs as opposed to WT-MT mice (Fig. 2 K). In summary, *Tet2*-deficient myeloid cells may accelerate liver fibrosis by promoting the intrahepatic expansion and pro-inflammatory polarization of *Tet2*^−/−^ MDMs.

### MDMs depletion attenuates liver fibrosis in *Tet2*^ΔMye^-CCl_4_ mice

**Figure 3.**
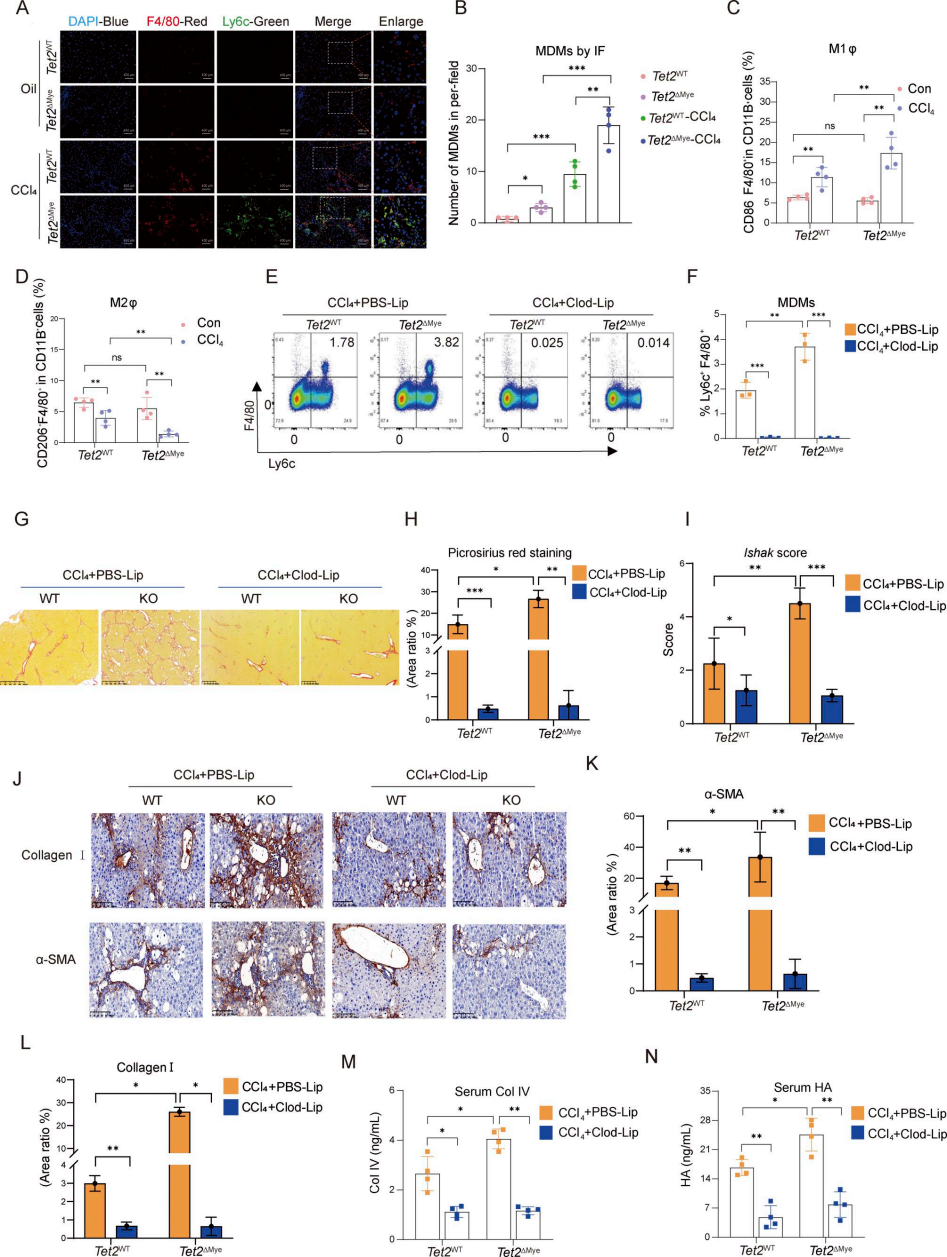
**MDMs depletion effectively inhibits liver fibrosis progression in *Tet2*** ^**ΔMye**^ **mice. (A and B)** IF assay (A) (scale bar, 400 μm) and statistical analysis (B) of MDMs infiltration in livers of oil- or CCl_4_-treated *Tet2*^WT^ and *Tet2*^ΔMye^ mice. Blue: DAPI, red: anti-F4/80, and green: anti-Ly6c (*n* = 4 for each group). **(C and D)** Frequency of CD86^+^ MDMs (M1) (C) and CD206^+^ MDMs (M2) (D) evaluated by flow cytometry in livers of oil- or CCl_4_-treated *Tet2*^WT^ and *Tet2*^ΔMye^ mice (*n* = 4 for each group). **(E and F)** Representative flow cytometry graphs (E) and statistical analysis (F) the of MDMs frequency in *Tet2*^WT^ and *Tet2*^ΔMye^ mice after treatment with PBS-Lip or Clod-Lip (*n* = 3 for each group). **(G–I)** Picrosirius red staining (G and H) (scale bar, 125 μm) and Ishak score (I) in livers of PBS-Lip– and Clod-Lip–treated *Tet2*^WT^ and *Tet2*^ΔMye^ mice with liver fibrosis. **(J–L)** IHC staining (J) and statistical analysis of α-SMA (K) and collagen I (L) expression in livers (scale bar, 100 μm) of PBS-Lip– and Clod-Lip–treated *Tet2*^WT^ and *Tet2*^ΔMye^ mice (*n* = 3 for each group). **(M and N)** Changes in serum Col IV (M) and HA (N) in CCl_4_-treated *Tet2*^WT^ and *Tet2*^ΔMye^ mice after PBS-Lip or Clod-Lip treatment (*n* = 4 for each group). Data are representative of at least two independent experiments with similar results (A–N). All data are shown as mean ± SD and were analyzed by two-way ANOVA with Sidak’s multiple comparison test (B–D, F, H, I, and K–N). ***P < 0.001; **P < 0.01; *P < 0.05; P > 0.05 not significant (ns).

In line with the expansion of *Tet2*^−/−^pMDMs in chimeric mice, immunofluorescence (IF) analysis revealed significantly greater intrahepatic MDMs accumulation in *Tet2*^ΔMye^-CCl_4_ mice compared with *Tet2*^WT^-CCl_4_ littermates (Fig. 3, A and B). Flow cytometry and IF analysis further demonstrated an elevated frequency of M1-like pMDMs and a reduced proportion of M2-like MDMs in *Tet2*^ΔMye^-CCl_4_ mice (Fig. 3, C and D; and Fig. S2, F–H).

To explore whether intrahepatic pMDMs expansion was the trigger of liver fibrosis progression in *Tet2*^ΔMye^-CCl_4_ mice, we administered clodronate liposomes (Clod-Lip) to deplete MDMs *in vivo*, with PBS liposomes (PBS-Lip) serving as a control (Fig. S2 I). Clod-Lip treatment effectively reduced the MDMs population (Fig. 3, E and F) and significantly attenuated collagen deposition, as evidenced by Picrosirius red staining and Ishak scoring (Fig. 3, G–I). Immune cell infiltration was markedly reduced in Clod-Lip–treated *Tet2*^ΔMye^-CCl_4_ mice, reaching levels comparable with those in *Tet2*^WT^-CCl_4_ controls (Fig. S2 J). Furthermore, Clod-Lip treatment normalized the expression of fibrotic markers, including α-SMA and collagen I (Fig. 3, J–L), and reduced serum levels of Col IV (Fig. 3 M) and HA (Fig. 3 N) in *Tet2*^ΔMye^-CCl_4_ mice. In conclusion, depletion of MDMs effectively ameliorates liver fibrosis in *Tet2*^ΔMye^-CCl_4_ mice, underscoring the critical role of intrahepatic pMDMs in driving liver fibrosis progression.

### *Tet2* ^ΔMye^ may drive MDMs infiltration via autonomous Ccl2 and Ccl8 production

**Figure 4.**
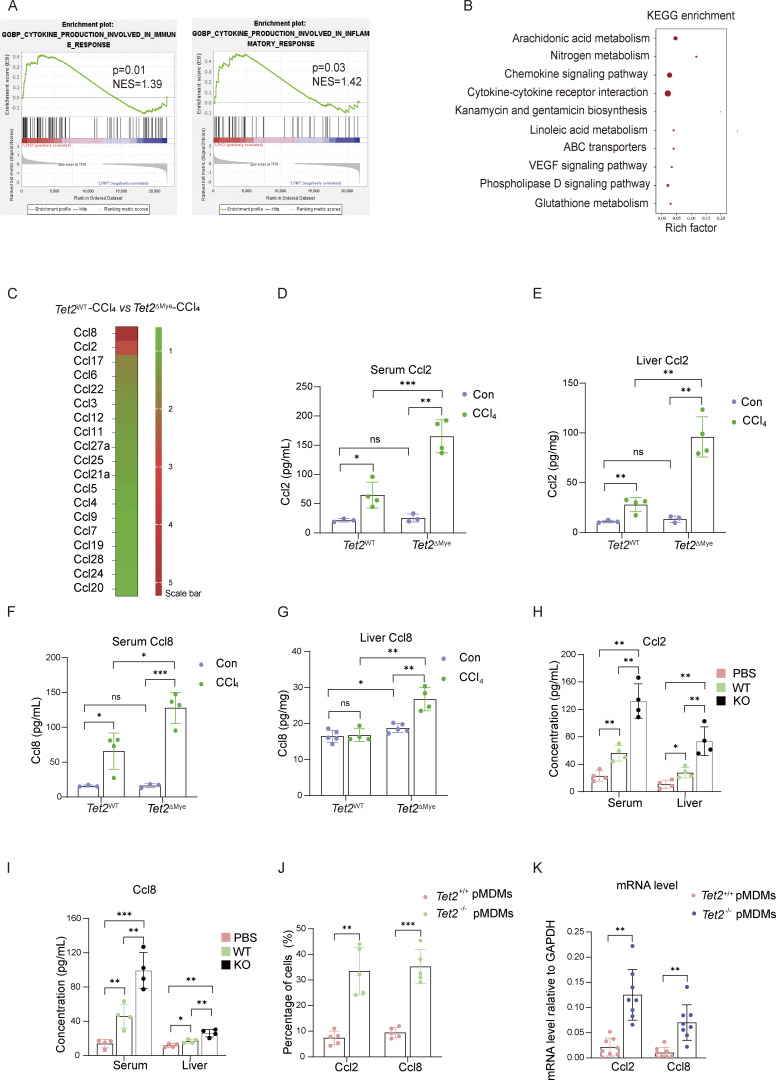
**The upregulated Ccl2 and Ccl8 secreted by *Tet2*** ^**−/−**^ **MDMs promote MDMs intrahepatic accumulation in a positive feedback manner. (A)** GSEA for cytokine production involved in immune response and cytokine production involved in inflammatory response signaling pathways in *Tet2*^WT^-CCl_4_ versus *Tet2*^ΔMye^-CCl_4_ littermates (*n* = 4–5 for each group). **(B)** KEGG analysis of DEGs in *Tet2*^WT^-CCl_4_ versus *Tet2*^ΔMye^-CCl_4_ littermates. **(C)** Expression profiles of C–C motif ligands detected by RNA sequence in livers of *Tet2*^WT^-CCl_4_ and *Tet2*^ΔMye^-CCl_4_ littermates (*n* = 5 for each group). **(D and E)** The effect of *Tet2*^ΔMye^ on Ccl2 levels in serum (D) and livers (E) of mice with or without liver fibrosis. pg/mg: Ccl2 levels per milligram of liver tissue (*n* = 4 for each group). **(F and G)** The Ccl8 levels in serum (F) and livers (G) of four groups of mice. pg/mg: Ccl8 levels per milligram of liver tissue (*n* = 4 for each group). **(H and I)** Changes in Ccl2 (H) and Ccl8 (I) levels in serum of scramble, WT-MT, and KO-MT mice (*n* = 4 for each group). **(K)** Detection of Ccl2 and Ccl8 expression in *Tet2*^+/+^ and *Tet2*^−/−^ MDMs by flow cytometry (*n* = 5 for each group) and RT-PCR (*n* = 8 for each group). Data are the accumulative results from at least two independent experiments (K) or are representative of at least two independent experiments with similar results (D–J). All data are shown as mean ± SD and were analyzed by two-way ANOVA with Sidak’s multiple comparison test (B–G, J, and K) or one-way ANOVA with Tukey’s multiple comparison test (H and I). ***P < 0.001; **P < 0.01; *P < 0.05; P > 0.05 not significant (ns).

**Figure S3.**
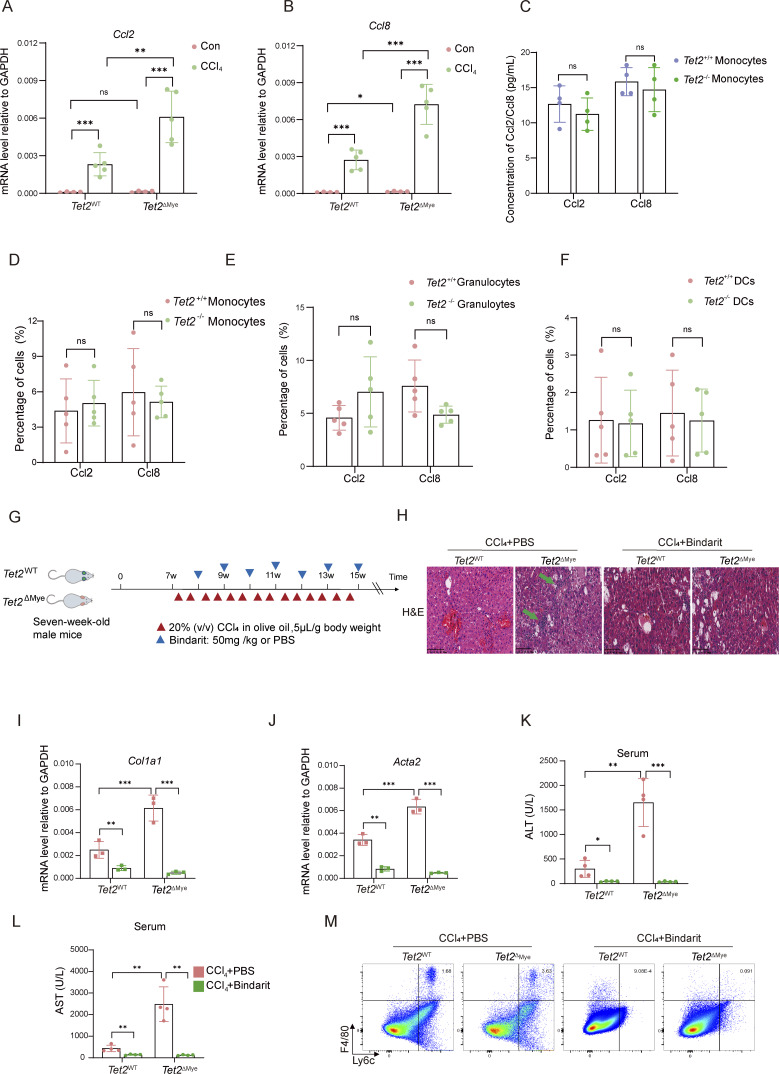
**Expression of Ccl2 and Ccl8 in *Tet2*** ^**WT**^ **-CCl**_**4**_ **and *Tet2*** ^**ΔMye**^ **-CCl**_**4**_ **mice and the effect of CCL2 and CCL8 inhibition on liver fibrosis progression. (A and B)** Changes in (A) *Ccl2* and (B) *Ccl8* mRNA levels detected by RT-PCR in liver tissues of *Tet2*^WT^-CCl_4_ and *Tet2*^ΔMye^-CCl_4_ mice (*Tet2*^WT^, *n* = 4; *Tet2*^ΔMye^, *n* = 4; *Tet2*^WT^-CCl_4_, *n* = 5; *Tet2*^ΔMye^-CCl_4_, *n* = 5). **(C–F)** Detection of CCL2 and CCL8 level in *Tet2*^+/+^and *Tet2*^−/−^ monocytes (C and D), granulocytes (E), and DCs (F) (*n* = 5 for each group). **(G)** Treatment schedule of Bindarit or PBS for *Tet2*^WT^-CCl_4_ and *Tet2*^ΔMye^-CCl_4_ mice. **(H)** Immune cell infiltration and the pathological structure of livers were evaluated by H&E staining for PBS and Bindarit in *Tet2*^WT^-CCl_4_ and *Tet2*^ΔMye^-CCl_4_ mice (*n* = 3). Green arrows indicate infiltrated immune cells in liver tissues. Scale bar, 100 μm. **(I and J)** mRNA levels of *Col1a1* (I) and *Acta2* (J) in livers of *Tet2*^WT^-CCl_4_ and *Tet2*^ΔMye^-CCl_4_ mice treated with PBS or Bindarit (*n* = 3 for each group). **(K and L)** Changes in serum ALT (K) and AST (L) after Bindarit treatment in *Tet2*^WT^-CCl_4_ and *Tet2*^ΔMye^-CCl_4_ mice (*n* = 4 for each group). **(M)** Representative flow cytometry graphs of MDMs in liver tissues of *Tet2*^WT^-CCl_4_ and *Tet2*^ΔMye^-CCl_4_ mice treated with PBS or Bindarit. Data are representative of at least two independent experiments with similar results (A–F and I–L). All data are shown as mean ± SD and were analyzed by two-tailed, paired Student’s *t* test (C–F) or two-way ANOVA with Sidak’s multiple comparison test (A, B, and I–L). ***P < 0.001; **P < 0.01; *P < 0.05; P > 0.05 not significant (ns).

To identify factors regulating MDMs intrahepatic infiltration in *Tet2*^ΔMye^-CCl_4_ mice, we performed RNA sequencing of liver tissues. Gene expression analysis revealed 130 and 134 differentially expressed genes (DEGs) in *Tet2*^WT^ vs. *Tet2*^ΔMye^ and *Tet2*^WT^-CCl_4_ vs. *Tet2*^ΔMye^-CCl_4_ comparisons, respectively (Fig. S2 K). Because of the significant difference of liver fibrosis severity between *Tet2*^WT^-CCl_4_ and *Tet2*^ΔMye^-CCl_4_ mice, we next focused on DEGs between the two groups. Gene set enrichment analysis (GSEA) highlighted significant enrichment of pathways related to “cytokine production involved in immune response” and “cytokine production involved in inflammatory response” in *Tet2*^ΔMye^-CCl_4_ mice (Fig. 4 A). KEGG analysis further confirmed the enrichment of “cytokine–cytokine receptor interaction” and “chemokine signaling pathway” (Fig. 4 B). Among chemokine (C–C motif) ligands, Ccl2 and Ccl8 were the most upregulated in *Tet2*^ΔMye^-CCl_4_ mice (Fig. 4 C), a finding validated using RT-PCR (Fig. S3, A and B). Enzyme-linked immunosorbent assays (ELISA) showed elevated levels of Ccl2 and Ccl8 in both serum and liver tissues of *Tet2*^ΔMye^-CCl_4_ mice in comparison with *Tet2*^WT^-CCl_4_ and control groups (Fig. 4, D–G). Similarly, we detected increased Ccl2 and Ccl8 in the serum of the KO-MT chimeric mice compared with WT-MT mice (Fig. 4, H and I).

Given the pivotal role of Ccl2 and Ccl8 in monocyte recruitment and MDMs accumulation, we sought to identify their cellular origins. ELISA and flow cytometry analysis revealed that CD45.2^+^*Tet2*^−/−^ monocytes, granulocytes, and dendritic cells exhibited similar Ccl2 and Ccl8 levels to *Tet2*^+/+^ counterparts (Fig. S3, C–F). In contrast, intrahepatic CD45.2^+^*Tet2*^−/−^ MDMs showed significantly higher Ccl2 and Ccl8 expression than that of *Tet2*^+/+^ MDMs (Fig. 4 J), indicating that *Tet2*^−/−^ MDMs are the primary source of these chemokines. Consistent with this, RT–quantitative PCR (qPCR) confirmed elevated *Ccl2* and *Ccl8* mRNA levels in *Tet2*^−/−^ MDMs isolated from *Tet2*^ΔMye^ mice compared with littermate controls (Fig. 4 K).

### Inhibition of Ccl2 and Ccl8 depletes MDMs and improves liver fibrosis in *Tet2*^ΔMye^-CCl_4_ mice

**Figure 5.**
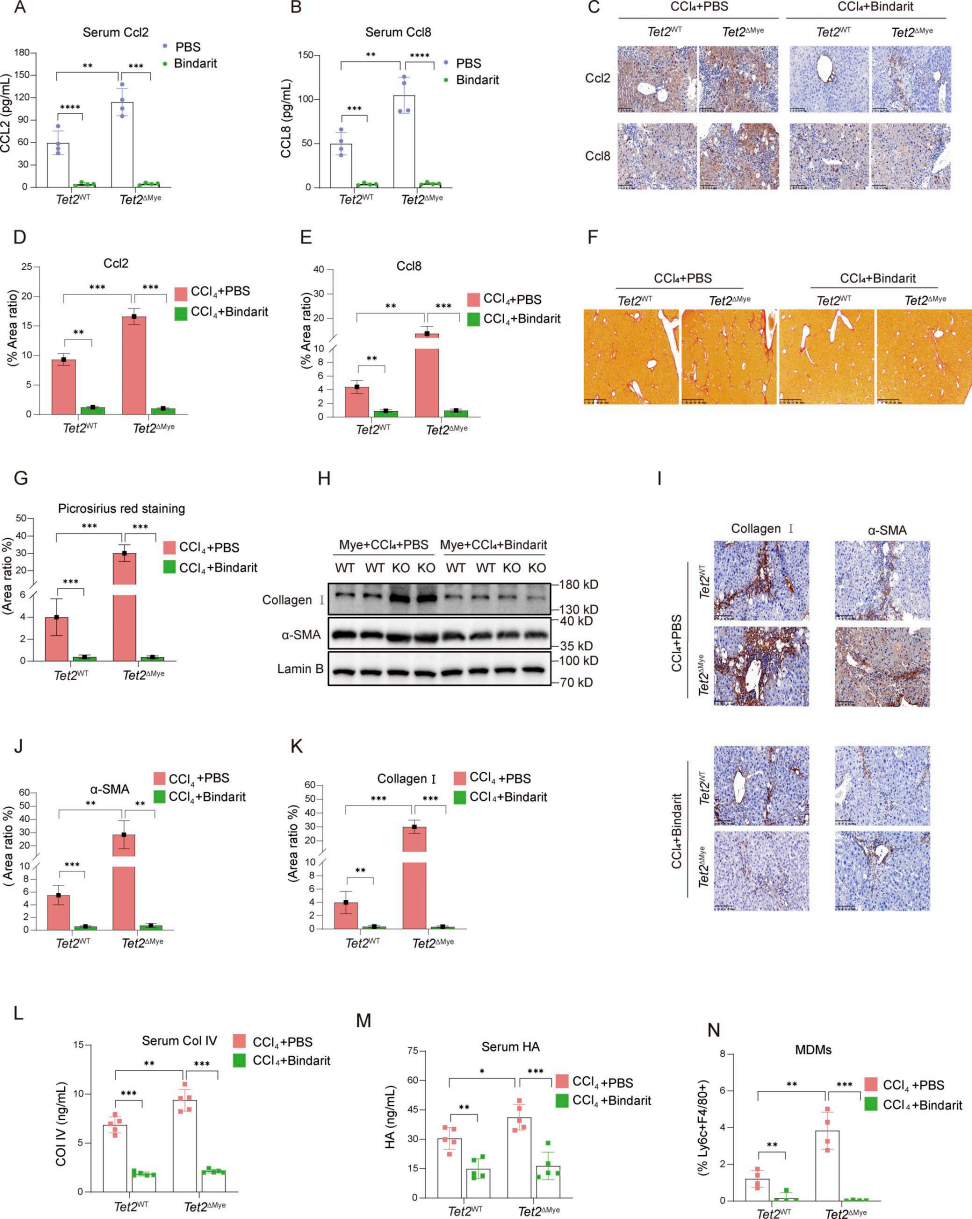
**Inhibition of Ccl2 and Ccl8 depletes MDMs and alleviates liver fibrosis in *Tet2*** ^**ΔMye**^ **-CCl**_**4**_ **mice. (A and B)** Effect of Bindarit inhibition on serum Ccl2 (A) and Ccl8 (B) levels in *Tet2*^WT^-CCl_4_ and *Tet2*^ΔMye^-CCl_4_ littermates (*n* = 4 for each group). **(C–E)** IHC staining (C) and statistical analysis of Ccl2 (D) and Ccl8 (E) expression after Bindarit treatment in livers of *Tet2*^WT^-CCl_4_ and *Tet2*^ΔMye^-CCl_4_ littermates (*n* = 4 for each group). Scale bar, 100 μm. **(F and G)** Picrosirius red staining (F) and statistical analysis (G) of collagen deposition in livers of *Tet2*^WT^-CCl_4_ and *Tet2*^ΔMye^-CCl_4_ littermates (*n* = 4 for each group). Scale bar, 100 μm. **(H–K)** Effect of Bindarit on α-SMA and collagen I expression evaluated by western blot (H) and IHC staining (I, J, and K) (scale bar, 100 μm) in livers of *Tet2*^WT^-CCl_4_ and *Tet2*^ΔMye^-CCl_4_ littermates (*n* = 4 for each group). **(L and M)** Changes in serum Col IV (M) and HA (N) in *Tet2*^WT^-CCl_4_ and *Tet2*^ΔMye^-CCl_4_ littermates after PBS or Bindarit treatment (*n* = 5 for each group). **(N)** Changes in intrahepatic MDMs frequency after Bindarit treatment in *Tet2*^WT^-CCl_4_ and *Tet2*^ΔMye^-CCl_4_ littermates. Data are representative of at least two independent experiments with similar results (A–L). All data are shown as mean ± SD and were analyzed by two-way ANOVA with Sidak’s multiple comparison test (A, B, D, E, G, and J–N). ****P < 0.0001; ***P < 0.001; **P < 0.01; *P < 0.05; P > 0.05 not significant (ns). Source data are available for this figure: SourceData F5.

To investigate the role of Ccl2 and Ccl8 in pMDMs intrahepatic infiltration and liver fibrosis progression, we treated mice with Bindarit, a dual inhibitor of Ccl2 and Ccl8, during the construction of liver fibrosis model in *Tet2*^ΔMye^ mice (Fig. S3 G). Bindarit treatment significantly reduced Ccl2 and Ccl8 levels in both serum and liver tissues of *Tet2*^WT^-CCl_4_ and *Tet2*^ΔMye^-CCl_4_ mice (Fig. 5, A–E). Picrosirius red staining revealed that Bindarit treatment markedly decreased collagen deposition, Ishak scores, and immune cell infiltration in *Tet2*^ΔMye^-CCl_4_ mice to levels comparable with those in *Tet2*^WT^-CCl_4_ mice (Fig. 5, F–H; and Fig. S3 H). Furthermore, Bindarit suppressed the expression of α-SMA and collagen I at both protein and mRNA levels in the liver (Fig. 5, I–K; and Fig. S3, I and J) and normalized serum levels of Col IV (Fig. 5 L), HA (Fig. 5 M), ALT (Fig. S3 K), and AST (Fig. S3 L) in *Tet2*^ΔMye^-CCl_4_ mice. Notably, Bindarit treatment also significantly reduced intrahepatic MDMs accumulation (Fig. 5 N and Fig. S3 M). Inhibition of Ccl2 and Ccl8 by Bindarit effectively suppresses MDMs intrahepatic infiltration and ameliorates liver fibrosis in *Tet2*^ΔMye^-CCl_4_ mice, highlighting the critical role of these chemokines in fibrotic progression.

### Ccl2/Ccl8-dependent Ccr2/Ccr3 activation promotes *Tet2*^−/−^ monocyte recruitment and pMDMs hepatic accumulation

**Figure 6.**
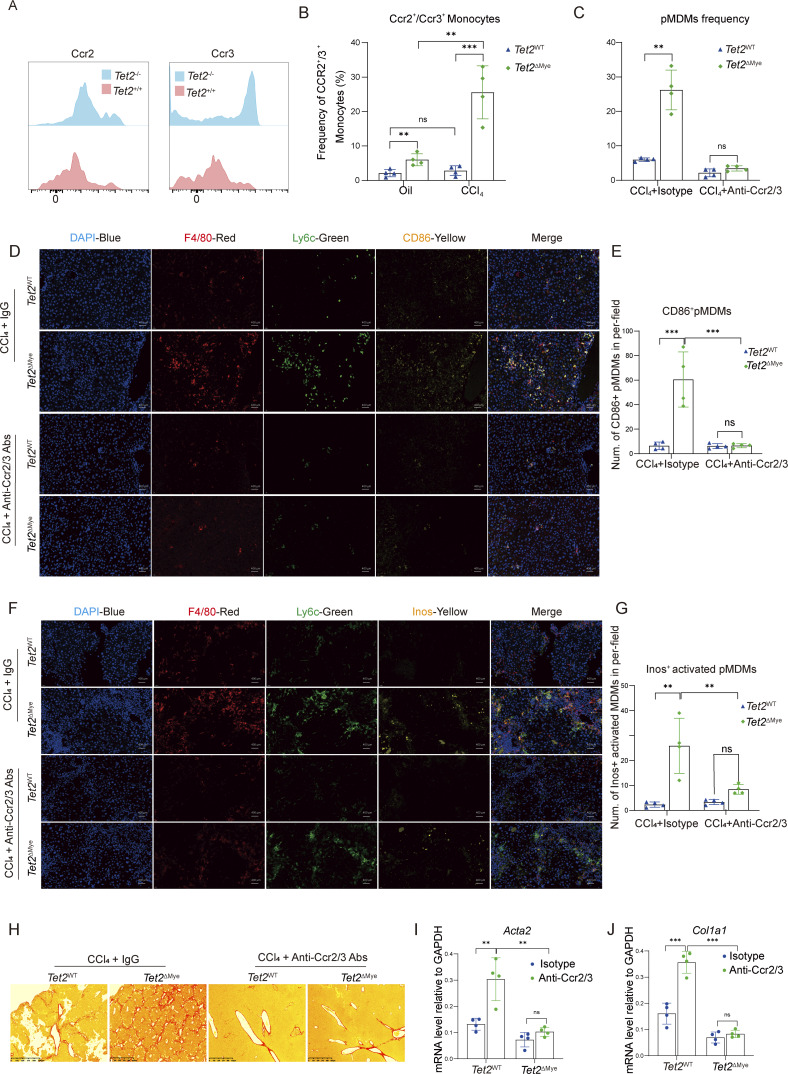
**Ccl2/Ccl8-dependent Ccr2/Ccr3 activation drives *Tet2*** ^***−/−***^ **monocyte recruitment and pMDMs accumulation in liver. (A)** Expression of Ccr2 and Ccr3 on *Tet2*^+/+^ and *Tet2*^−/−^ monocytes isolated from *Tet2*^WT^ and *Tet2*^ΔMye^ mice (*n* = 4 for each group). **(B)** Frequency of Ccr2^+^ and Ccr3^+^ monocytes in oil- and CCl_4_-treated *Tet2*^WT^ and *Tet2*^ΔMye^ mice (*n* = 4 for each group). **(C)** Effect of anti-Ccr2/3 treatment on the frequency of pMDMs in CCl_4_-treated *Tet2*^WT^ and *Tet2*^ΔMye^ (*n* = 4 for each group). **(D and E)** CD86^+^ MDMs distribution (D) and statistical analysis (E) evaluated by IF staining after anti-Ccr2/3 Ab treatment (*n* = 4 for each group). Blue: DAPI, red: anti-F4/80, green: anti-Ly6c, and yellow: anti-CD206. Scale bar: 400 μm. **(F and G)** The distribution of Inos^+^ MDMs (activated MDMs) (F) and statistical analysis (G) after anti-Ccr2/3 Ab treatment (*n* = 4 for each group). Blue: DAPI, red: anti-F4/80, green: anti-Ly6c, and yellow: anti-Inos. Scale bar: 400 μm. **(H)** Change of collagen deposition evaluated by Picrosirius red staining in livers of IgG and anti-Ccr2/3–treated *Tet2*^WT^-CCl_4_ and *Tet2*^ΔMye^-CCl_4_ mice (*n* = 4 for each group). **(I and J)** mRNA levels of *Acta2* (I) and *Col1a1* (J) in liver tissues of IgG and anti-Ccr2/3–treated *Tet2*^WT^-CCl_4_ and *Tet2*^ΔMye^-CCl_4_ mice (*n* = 4 for each group). Data are representative of at least two independent experiments with similar results (A–J). All data are shown as mean ± SD and were analyzed by two-way ANOVA with Sidak’s multiple comparison test (B, C, E, G, I, and J). ***P < 0.001; **P < 0.01; *P < 0.05; P > 0.05 not significant (ns).

**Figure S4.**
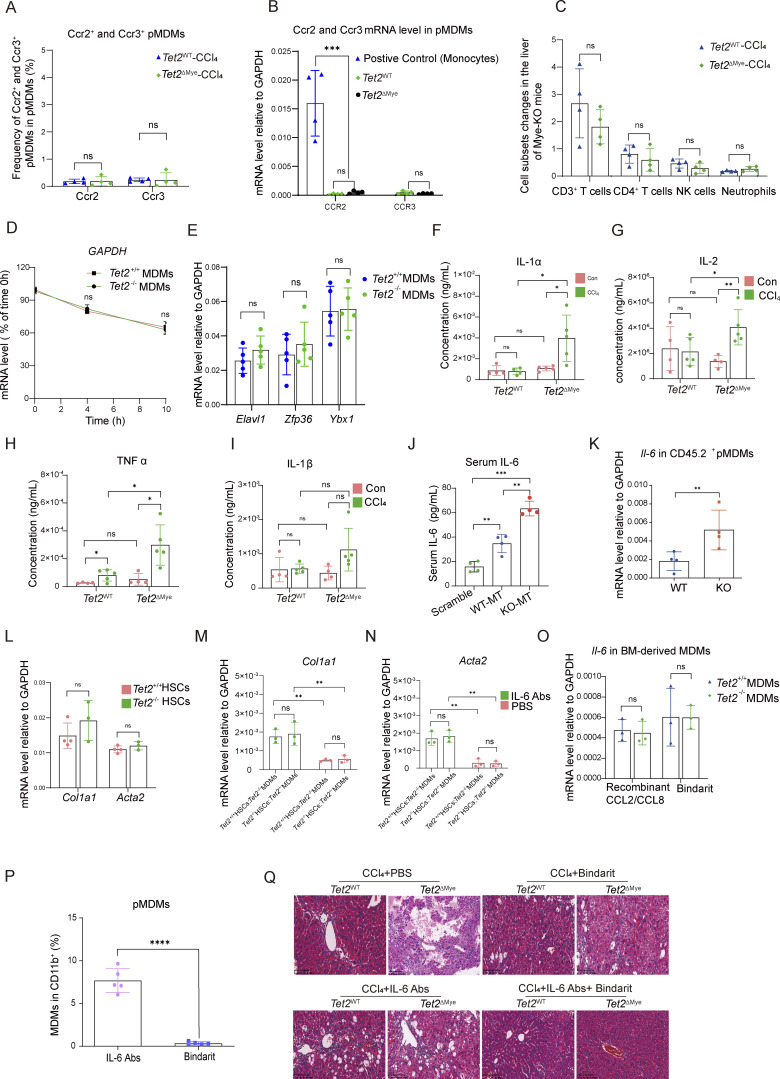
**Effect of *Tet2* deficiency on *Ccl2* and *Ccl8* mRNA stability and IL-6 neutralization on liver fibrosis progression. (A)** The frequency of Ccr2^+^ and Ccr3^+^ pMDMs in *Tet2*^WT^-CCl_4_ and *Tet2*^ΔMye^-CCl_4_ mice (*n* = 4 for each group). **(B)** Expression of Ccr2 and Ccr3 on monocytes, *Tet2*^+/+^ pMDMs, and *Tet2*^−/−^ pMDMs (*n* = 4 for each group). **(C)** The infiltration difference of other Ccr2 or Ccr3 expressing cell types in *Tet2*^WT^-CCl_4_ and *Tet2*^ΔMye^-CCl_4_ mice (*n* = 4 for each group). **(D)** GAPDH mRNA decay curve in *Tet2*^+/+^ pMDMs and *Tet2*^−/−^ pMDMs (*n* = 6 for each group). **(E)** Transcriptional level of Elavl1, Znf36, and Ybx1 in *Tet2*^+/+^ pMDMs and *Tet2*^−/−^ pMDMs (*n* = 4 for each group). **(F–I)** Serum levels of (F) IL-1α, (G) IL-2, (H) TNFα, and (I) IL-1β in livers of *Tet2*^WT^-CCl_4_ and *Tet2*^ΔMye^-CCl_4_ mice (*n* = 5 for each group). **(J)** Serum IL-6 levels in scramble, WT-MT, and KO-MT mice (*n* = 4 for each group). **(K)** Il-6 levels in CD45.2^+^ pMDMs isolated from livers of WT-MT and KO-MT mice (*n* = 4). **(L)** mRNA levels of *Acta2* and *Col1a1* in *Tet2*^+/+^and *Tet2*^−/−^ HSCs (*n* = 4 for each group). **(M and N)** Effect of anti–IL-6 Abs treatment on mRNA levels of *Col1a1* (M) and *Acta2* (N) in *Tet2*^+/+^and *Tet2*^−/−^ HSCs co-cultured with *Tet2*^+/+^or *Tet2*^−/−^ MDMs detected by RT-PCR *in vitro* (*n* = 3 for each group). **(O)** Effect on recombinant Ccl2 and Ccl8 on Il-6 expression in *Tet2*^+/+^ pMDMs and *Tet2*^−/−^ pMDMs (*n* = 3 for each group). **(P)** Detection of MDMs in livers by flow cytometry after Bindarit or IL-6 Abs treatment for 2 wk (*n* = 5 for each group). **(Q)** H&E staining of liver tissues treated with PBS, Bindarit, IL-6 Abs, or Bindarit plus IL-6 Abs in *Tet2*^WT^-CCl_4_ and *Tet2*^ΔMye^-CCl_4_ mice (*n* = 4 for each group). Data are representative of at least two independent experiments with similar results (A–P). All data are shown as mean ± SD and were analyzed by two-tailed, unpaired Student’s *t* test (A, C, D, E, K, L, and O) or one-way ANOVA with Tukey’s multiple comparison test (B and J) or two-way ANOVA with Sidak’s multiple comparison test (F–I, M, and N). ****P < 0.0001; ***P < 0.001; **P < 0.01; *P < 0.05; P > 0.05 not significant (ns).

Since MDMs were often originated from peripheral monocytes via chemokines signaling, we, therefore, hypothesized that *Tet2*^−/−^monocytes might exhibit superior capability to infiltrate into liver. Given the upregulation of Ccl2 and Ccl8, we firstly detected the expression of Ccr2 and Ccr3, the receptors of Ccl2 and Ccl8, on *Tet2*^−/−^ monocytes. Flow cytometric analysis revealed cell-autonomous upregulation of Ccr2 and Ccr3 in *Tet2*^−/−^ monocytes, with significant increases in both surface density and population frequency compared with *Tet2*^+/+^ counterparts (Fig. 6, A and B). Notably, we identified a pre-fibrotic phenotype in *Tet2*^−/−^ monocytes, characterized by baseline elevation of Ccr2^+^/Ccr3^+^ subpopulations, a phenotype that was substantially amplified following CCl_4_ challenge (Fig. 6 B). Notably, Ccr2 and Ccr3 expression was nearly undetectable in pMDMs compared with that in monocytes (Fig. S4, A and B). Therapeutic co-blockade of Ccr2/Ccr3 decreased the frequency of pMDMs in the liver of *Tet2*^ΔMye^-CCl_4_ mice (Fig. 6, C–E). IF staining identified the decreased Inos^+^ activated MDMs in liver (Fig. 6, F and G). Anti-Ccr2/Ccr3 effectively inhibited collagen deposition (Fig. 6 H), concomitant with suppressed *Acta2* and *Col1a1* expression (Fig. 6, I and J). To determine the cellular specificity of Ccl2- and Ccl8-dependent recruitment, we performed comprehensive flow cytometric profiling of Ccr2/Ccr3-expressing immune populations in fibrotic livers. *Tet2*-deficient livers showed no significant differences in hepatic infiltration frequencies of CD3^+^ T cells, CD4^+^ T cells, NK cells, or neutrophils compared with WT controls (Fig. S4 C). These data reveal a remarkable cellular selectivity, where *Tet2* deletion specifically enhances pMDMs recruitment without broadly affecting other Ccr2^+^ and Ccr3^+^ immune subsets. Collectively, *Tet2* deficiency primes monocytes for hyperresponsiveness to chemokine signaling through Ccr2/Ccr3 upregulation, which critically mediates the progression of fibrosis.

### *Tet2* deficiency enhances *Ccl2 and Ccl8* mRNA stability by modifying 5-hydroxymethylcytosine–dependent RNA–protein interactions

**Figure 7.**
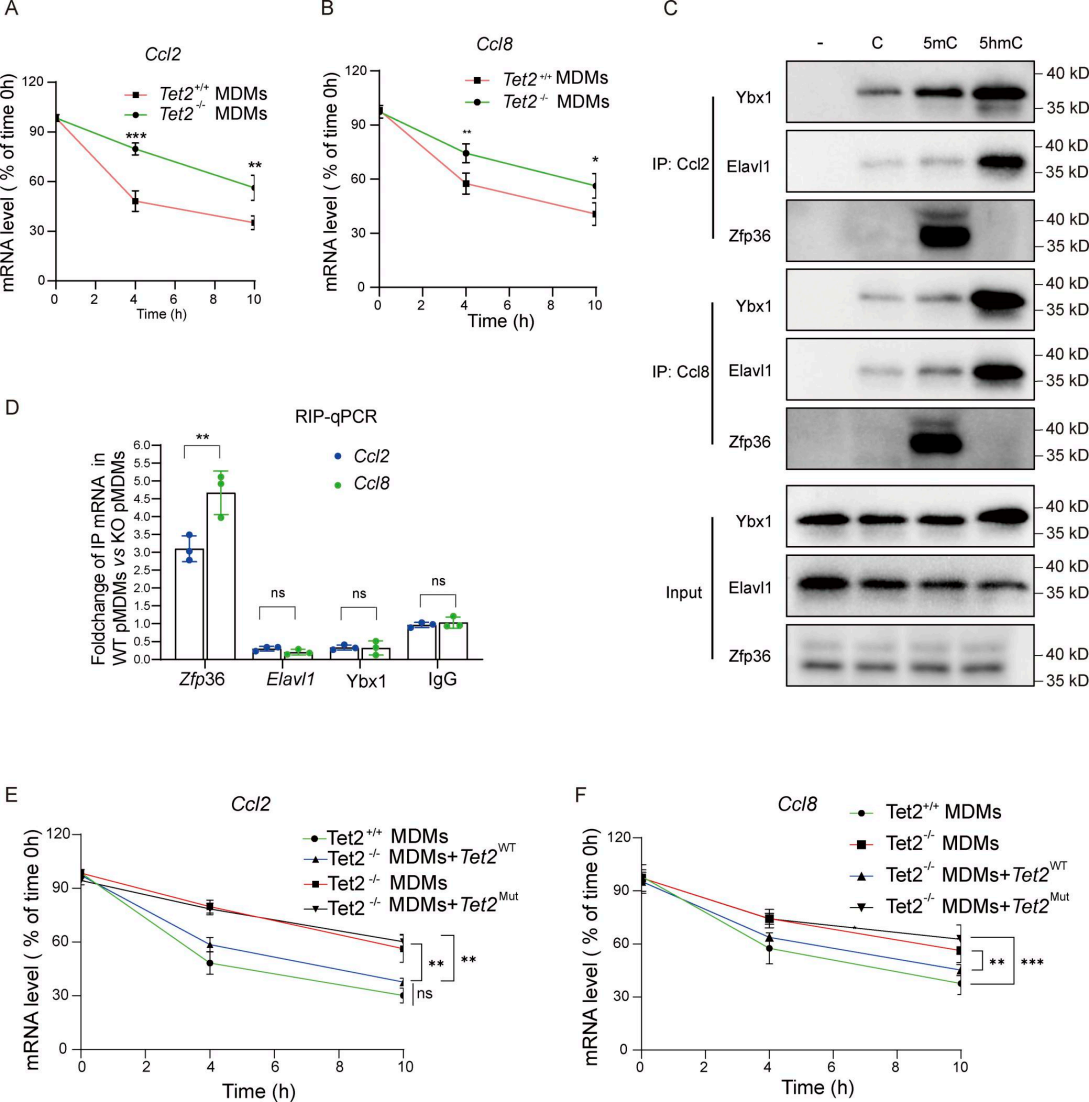
**Tet2 deficiency enhances *Ccl2* and *Ccl8* mRNA stability by modifying 5hmC-dependent RNA**–**protein interactions. (A and B)***Ccl2* (A) and *Ccl8* (B) mRNA decay in *Tet2*^+/+^ and *Tet2*^−/−^ MDMs (*n* = 6 for each group). **(C)***Tet2*-mediated oxidation of *Ccl2* and *Ccl8* mRNA 5mC disrupts its binding with Ybx1, Elavl1, and Zfp36. Pull-down assay was performed by incubating C, 5mC, and 5hmC oligos of *Ccl2* and *Ccl8* mRNA with cell lysate from THP1-derived pMDMs (*n* = 3 for each group). **(D)** Effect of *Tet2* deficiency on the binding enrichment of Ybx1, Elavl1, and Zfp36 at 3′UTR of *Ccl2* and *Ccl8* mRNA. Tet2-binding sites were mapped in the mRNA of *Ccl2* and *Ccl8* by qPCR of Ybx1, Elavl1, and Zfp36 RIP product in THP1-derived pMDMs (*n* = 3 for each group). **(E and F)** Effect of enzymatic inactivation of Tet2 via catalytic domain mutation on stabilization of *Ccl2* (F) and *Ccl8* (G) transcripts (*n* = 4 for each group). Data are the accumulative results from at least two independent experiments (A, B, D, E, and F) or are representative of at least two independent experiments with similar results (C and D). All data are shown as mean ± SD and were analyzed by two-tailed, unpaired Student’s *t* test (A, B, and D–F). ***P < 0.001; **P < 0.01; *P < 0.05; P > 0.05 not significant (ns). Source data are available for this figure: SourceData F7.

As a 5-methylcytosine (5mC) “eraser,” *Tet2* regulates mRNA stability by modulating AU-rich element (ARE)-mediated protein binding, while zinc finger protein 36 (*Zfp36*) promotes ARE-containing mRNA decay. Bioinformatics analysis of 3′ UTR sequences revealed that both *Ccl2 and Ccl8* harbor ARE motifs (Table S6). We determined that *Tet2* deficiency significantly enhances the stability of *Ccl2* and *Ccl8* mRNA (Fig. 7, A and B), but not *Gapdh* mRNA (Fig. S4 D), which may represent the molecular mechanism underlying the upregulation of Ccl2 and Ccl8 in pMDMs as well as the elevated levels of Ccl2 and Ccl8 observed in *Tet2*^ΔMye^-CCl_4_ mice. We therefore hypothesized that *Tet2* deficiency modulates the binding affinity of Y-box–binding protein 1 (Ybx1), Elavl1, and Zfp36 to *Ccl2* and *Ccl8* mRNAs by altering the methylation status of ARE regions in their 3′ UTRs, consequently influencing mRNA stability. We found that *Tet2* depletion did not alter the transcriptional levels of *Ybx1*, *Elavl1*, or *Zfp36* in THP1-derived pMDMs (Fig. S4 E). However, the oxidation of 5mC to 5-hydroxymethylcytosine (5hmC) in Ccl2 and Ccl8 mRNAs exerts bidirectional regulation on RNA–protein interactions: 5hmC modification significantly enhanced binding affinity of the stabilizing factors *Ybx1* and Elavl1, while potently inhibiting recruitment of the decay-promoting factor Zfp36 (Fig. 7 C). To further assess the functional consequences of *Tet2* deficiency on these regulatory interactions, we performed RNA immunoprecipitation (RIP)-qPCR in bone marrow cell (BMC)-derived pMDMs. Compared with WT controls, *Tet2*^−/−^ pMDMs exhibited substantially increased association of Ybx1 and Elavl1 with *Ccl2* and *Ccl8* mRNAs, while showing markedly reduced Zfp36 binding (Fig. 7 D). Consistent with those RNA-binding proteins binding shift, enzymatic inactivation of *Tet2* via catalytic domain mutation led to pronounced stabilization of *Ccl2* and *Ccl8* transcripts (Fig. 7, E and F). Our findings demonstrate that *Tet2* regulates the stability of *Ccl2* and *Ccl8* mRNAs in an enzyme activity-dependent manner by modulating the methylation landscape of ARE motifs in their 3′ UTRs.

### Upregulated IL-6 secreted by *Tet2*^−/−^ pMDMs activates HSCs in *Tet2*^ΔMye^-CCl_4_ Mice

**Figure 8.**
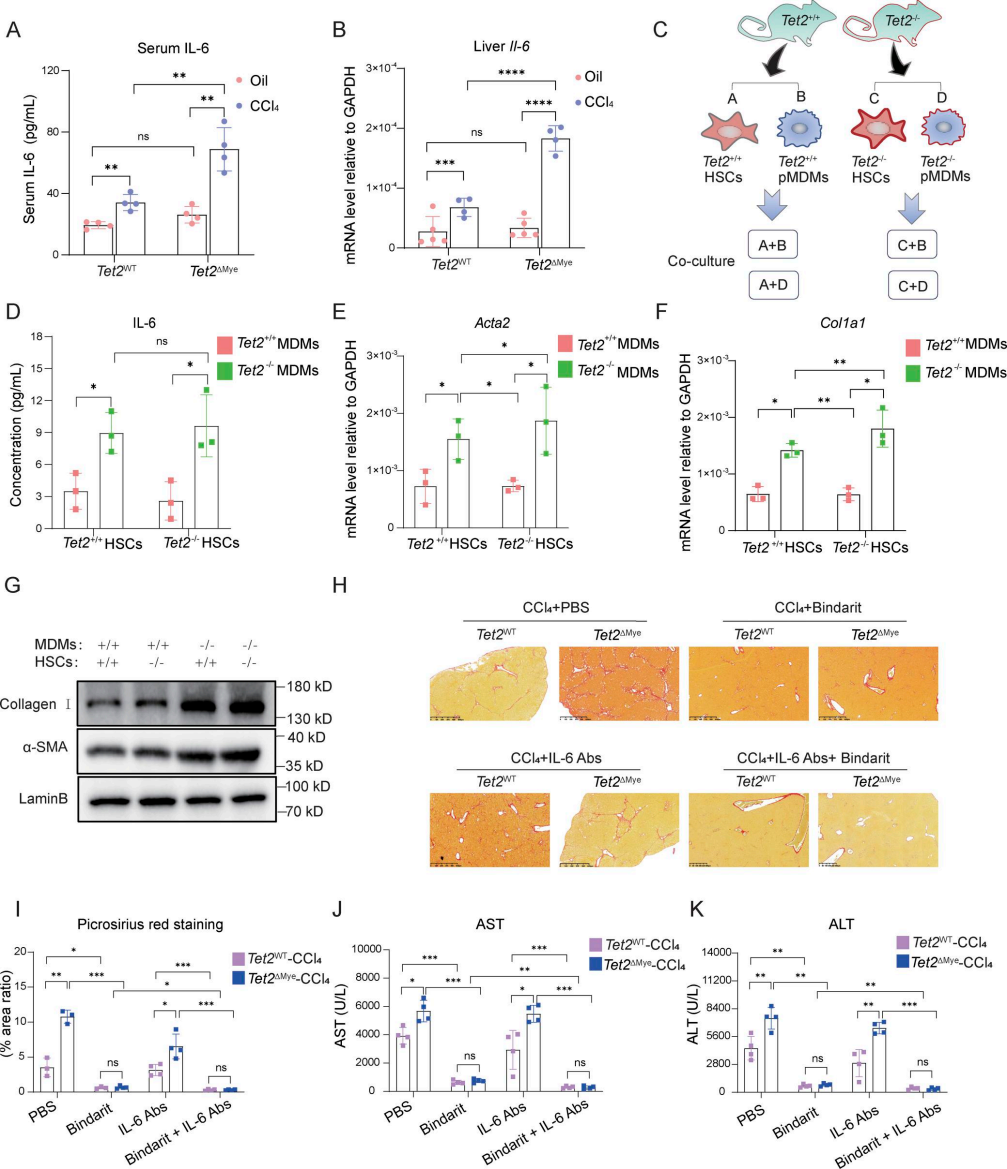
**The upregulating IL-6 secreted by *Tet2*** ^**−/−**^ **pMDMs activated HSCs in *Tet2*** ^**ΔMye**^ **-CCl**_**4**_ **mice. (A)** Effect of *Tet2*^ΔMye^ on serum IL-6 levels evaluated by ELISA in oil- or CCl_4_-treated *Tet2*^WT^ and *Tet2*^ΔMye^ mice (*n* = 4 for each group). **(B)** Effect of *Tet2*^ΔMye^ on *Il-6* mRNA levels in livers of oil- or CCl_4_-treated *Tet2*^WT^ and *Tet2*^ΔMye^ mice (*n* = 4 for oil-treated groups, *n* = 5 for CCl_4_-treated groups). **(C)** Experimental design of co-culture between *Tet2*^+/+^ or *Tet2*^−/−^ HSCs and *Tet2*^+/+^ or *Tet2*^−/−^ pMDMs. *Tet2*^+/+^ and *Tet2*^−/−^ pMDMs were isolated from livers of CCl_4_-treated *Tet2*^WT^ and *Tet2*^ΔMye^ mice. Naïve *Tet2*^+/+^ and *Tet2*^−/−^ HSCs were isolated from oil-treated *Tet2*^WT^ and *Tet2*^ΔMye^ mice. **(D)** Detection of IL-6 in the supernatant after co-culture by ELISA (*n* = 3 for each group). **(E–G)** mRNA and protein levels of *Acta2* (E and G) and *Col1a1* (F and G) detected by RT-PCR in *Tet2*^*+/+*^ and *Tet2*^−/−^ HSCs co-cultured with *Tet2*^*+/+*^ or *Tet2*^−/−^ pMDMs (*n* = 3 for each group). **(H and I)** Picrosirius red staining (H) (scale bar, 125 μm) and statistical analysis (I) of livers of oil- or CCl_4_-treated *Tet2*^WT^ and *Tet2*^ΔMye^ mice after Bindarit plus anti-IL-6 Abs treatment (*n* = 4 for each group). **(J and K)** Changes in serum ALT (J) and AST (K) levels in CCl_4_-treated *Tet2*^WT^ and *Tet2*^ΔMye^ mice after treatment with Bindarit, anti–IL-6 Abs, or Bindarit plus anti–IL-6 Abs (*n* = 4 for each group). Data are representative of at least two independent experiments with similar results (A–K). All data are shown as mean ± SD and were analyzed by two-way ANOVA with Sidak’s multiple comparison test (A, B, D–F, and I–K). ****P < 0.0001; ***P < 0.001; **P < 0.01; *P < 0.05; P > 0.05 not significant (ns). Source data are available for this figure: SourceData F8.

Because of the significant intrahepatic expansion of pMDMs in *Tet2*^ΔMye^-CCl_4_ mice, we assessed the levels of key inflammatory cytokines secreted by pMDMs to promote liver fibrosis progression. ELISA analysis revealed that IL-6 was the most markedly elevated cytokine in *Tet2*^ΔMye^-CCl_4_ mice compared with IL-1α, IL-2, and TNFα, whereas IL-1β levels remained unchanged (Fig. 8, A and B; and Fig. S4, F–I). Consistent with this, WT-MT chimeric mice also exhibited higher serum IL-6 levels than those KO-MT mice (Fig. S4 J), indicating that *Tet2*^−/−^ myeloid cells may be the primary source of elevated IL-6. Furthermore, we showed that *Tet2*^−/−^ pMDMs produced higher levels of IL-6 than *Tet2*^−/−^ pMDMs (Fig. S4 K).

To determine whether *Tet2*^−/−^ pMDMs activate HSCs through IL-6, we established an *in vitro* co-culture system of intrahepatic MDMs and HSCs (Fig. 8 C). *Tet2*^+/+^ and *Tet2*^−/−^ MDMs were isolated from CCl_4_-treated *Tet2*^WT^ and *Tet2*^ΔMye^ mice, whereas HSCs were isolated from oil-treated WT mice to exclude confounding effects of *Tet2* deficiency or CCl_4_ treatment on HSCs properties. Notably, *Tet2* deficiency did not significantly affect HSCs activation (Fig. S4 L). In co-culture, *Tet2*^−/−^ MDMs secreted higher levels of IL-6 than *Tet2*^−/−^ MDMs (Fig. 8 D), and only HSCs co-cultured with *Tet2*^−/−^ pMDMs exhibited increased expression of α-SMA and *Col1a1*, indicating HSCs activation (Fig. 8, E–G). Furthermore, IL-6 neutralization using anti–IL-6 antibodies (Abs) significantly reduced α-SMA and *Col1a1* expression in HSCs co-cultured with *Tet2*^−/−^ pMDMs (Fig. S4, M and N), confirming the role of *Il-6* in HSCs activation. To investigate whether elevated intrahepatic Ccl2 and Ccl8 regulates IL-6 expression in pMDMs, we treated pMDMs isolated from *Tet2*^ΔMye^-CCl_4_ mice with recombinant Ccl2 and Ccl8. The results showed that neither chemokine significantly altered IL-6 expression in pMDMs (Fig. S4 O).

To evaluate the therapeutic potential of IL-6 blockade, we administered anti-mouse IL-6 Abs to *Tet2*^ΔMye^-CCl_4_ mice. Although IL-6 neutralization alone modestly reduced collagen deposition (Fig. 8, H and I) and serum AST (Fig. 8 J) and ALT (Fig. 8 K) levels, it was less effective than Bindarit treatment. Notably, IL-6 Abs did not significantly reduce pMDMs frequency in the liver compared with Bindarit treatment (Fig. S4 P). However, combining Bindarit with IL-6 Abs more effectively attenuated collagen deposition, serum AST and ALT levels, and immune cell infiltration compared with either treatment alone (Fig. 8, H–K; and Fig. S4 Q). Therefore, *Tet2*^−/−^ pMDMs secrete elevated IL-6, which promotes HSCs activation and contributes to liver fibrosis. Combining MDMs depletion with IL-6 blockade represents a more effective therapeutic strategy for mitigating fibrosis in *Tet2*^ΔMye^-CCl_4_ mice.

### *Tet2* ^ΔMye^-induced myeloid hematopoiesis in aging host accelerates liver fibrosis

**Figure 9.**
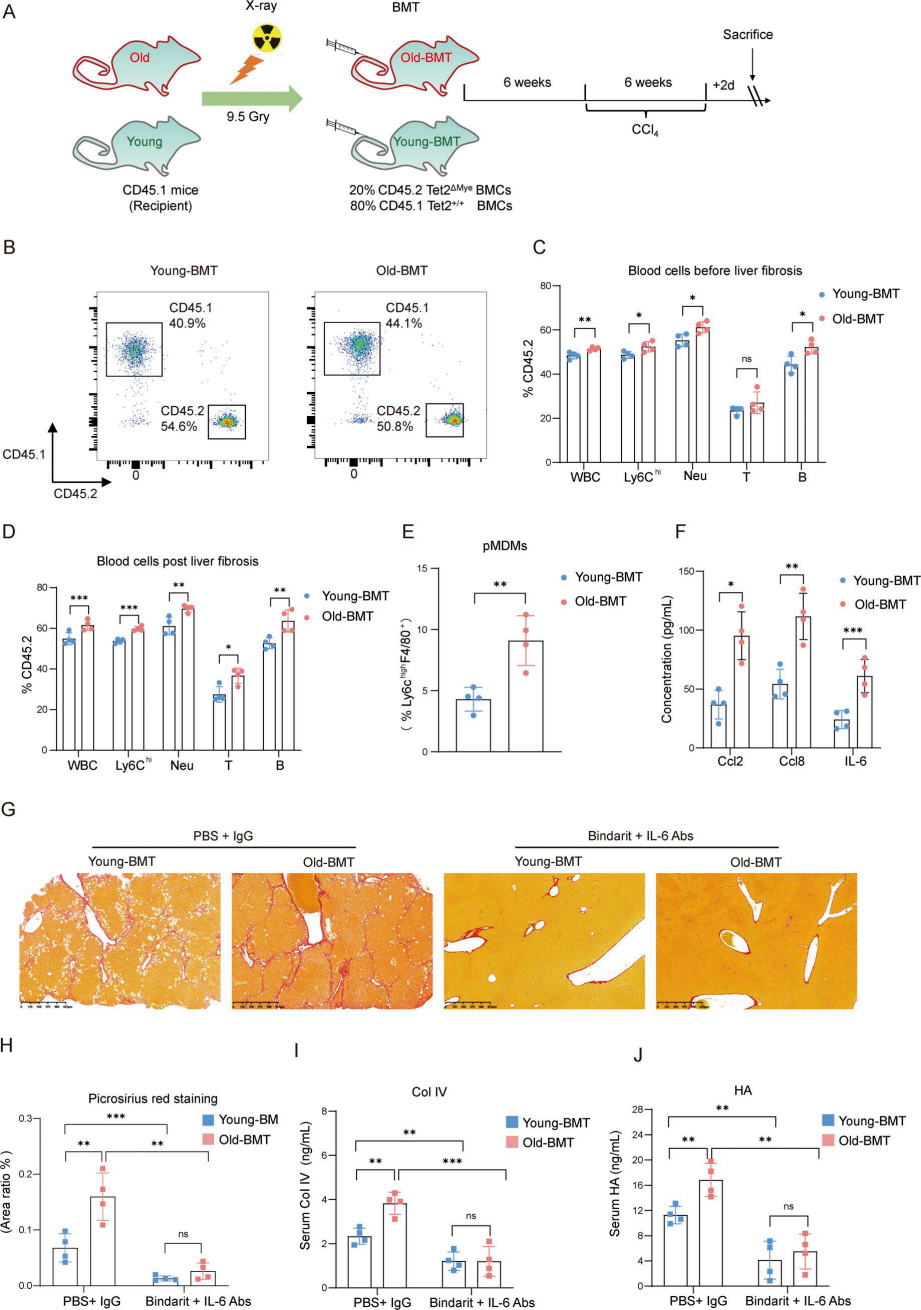
***Tet2*** ^**ΔMye**^ **-induced myeloid hematopoiesis in aging mice accelerate liver fibrosis. (A)** A chimeric mouse model of *Tet2*^ΔMye^-induced myeloid hematopoiesis was constructed in young and aged mice. CCl_4_ was administered to induce liver fibrosis in young-BMT and old-BMT mice. **(B)** The profiles the reconstruction of CD45.2^+^*Tet2*^−/−^ BMCs in the peripheral blood of young-BMT and old-BMT mice (*n* = 4 for each group). **(C and D)** Blood cell composition of CD45.2^+^ cells in young-BMT and old-BMT mice with (C) or without (D) CCl_4_-induced liver fibrosis (*n* = 4 for each group). **(E)** Frequency of pMDMs in livers of young-BMT and old-BMT mice with CCl_4_-induced liver fibrosis (*n* = 4 for each group). **(F)** The serum levels of Ccl2, Ccl8, and IL-6 in young-BMT and old-BMT mice with liver fibrosis (*n* = 4 for each group). **(G and H)** Picrosirius red staining (G) (scale bar, 125 μm) and statistical analysis (H) of collagen deposition in livers of CCl_4_-treated young-BMT and old-BMT mice after Bindarit plus anti–IL-6 Abs treatment (*n* = 4 for each group). **(I and J)** Changes in serum Col IV (I) and HA (J) levels in young-BMT and old-BMT mice treated with Bindarit, anti–IL-6 Abs, or Bindarit plus anti–IL-6 Abs. Data are representative of at least two independent experiments with similar results (A–J). All data are shown as mean ± SD and were analyzed by two-tailed, unpaired Student’s *t* test (C–F), or two-way ANOVA with Sidak’s multiple comparison test (H–J). ***P < 0.001; **P < 0.01; *P < 0.05; P > 0.05 not significant (ns).

*TET2* mutations, frequently observed in myeloid cells of the elderly, drive myeloid expansion. To investigate whether *Tet2*^ΔMye^ in aging hosts exacerbates liver fibrosis through the Ccl2/Ccl8–pMDMs–HSCs axis, we established a competitive bone marrow transplantation (BMT) model using *Tet2*-deficient BMCs (Fig. 9 A). CD45.1^+^*Tet2*^−/−^ BMCs (80%) and CD45.2^+^*Tet2*^ΔMye^ BMCs (20%) were transplanted into lethally irradiated young (young-BMT) and old (old-BMT) CD45.1 mice. By 6 wk after transplantation, CD45.2^+^ cells constituted ∼50% of peripheral blood leukocytes in both groups (Fig. 9 B). CD45.2^+^*Tet2*^−/−^ leukocytes, Ly6c^high^ monocytes, and neutrophils exhibited significant expansion, with greater expansion in old-BMT mice than in young-BMT mice (Fig. 9 C), recapitulating myeloid hematopoiesis observed in elderly individuals with *TET2* mutations.

To evaluate whether *Tet2*^ΔMye^-induced myeloid hematopoiesis increased the risk of liver fibrosis in old-BMT mice, young-BMT and old-BMT mice were subjected to CCl_4_-induced liver fibrosis. Old-BMT mice displayed higher levels of *Tet2*^−/−^ Ly6c^high^ monocytes (Fig. 9 D); intrahepatic pMDMs (Fig. 9 E); and serum Ccl2, Ccl8, and IL-6 (Fig. 9 F) compared with young-BMT mice. Combined treatment with Bindarit and IL-6 Abs effectively attenuated fibrosis, reducing collagen deposition (Fig. 9, G and H), serum Col IV (Fig. 9 I), and HA levels (Fig. 9 J) in old-BMT mice.

**Figure S5.**
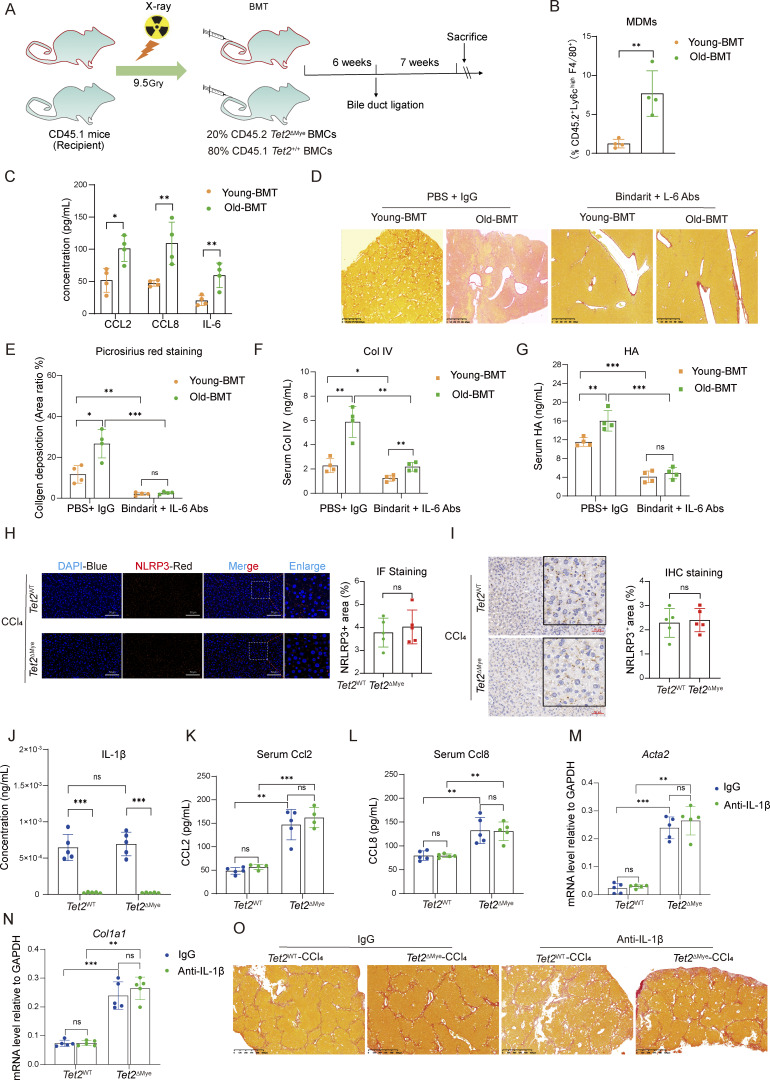
**Aging-related *Tet2*** ^**ΔMye**^ **-driven clonal hematopoiesis exacerbates liver fibrosis, and IL-1β–NLRP3 pathway is not involved in the liver fibrosis in Mye-KO-CCl**_**4**_ **mice. (A)** Chart for the construction of an aging-related *Tet2*^ΔMye^-induced clonal hematopoiesis mouse model with liver fibrosis. BDL was performed to construct a liver fibrosis model in young-BMT and old-BMT mice 6 wk after BMT. **(B)** Frequency of MDMs in livers of young-BMT and old-BMT mice after BDL for 4 wk (*n* = 4 for each group). **(C)** The serum levels of CCL2, CCL8, and IL-6 in young-BMT and old-BMT mice after BDL for 4 wk (*n* = 4 for each group). **(D and E)** Picrosirius red staining (D) (scale bar, 125 μm) and quantitative analysis of collagen deposition (E) in livers from young-BMT and old-BMT mice following BDL, treated with Bindarit plus anti–IL-6 Abs (*n* = 4 for each group). **(F and G)** Changes of serum Col IV (F) and HA (G) levels in young-BMT and old-BMT mice treated with Bindarit, anti–IL-6 Abs, or Bindarit plus anti–IL-6 Abs (*n* = 4 for each group). **(H)** IF (scale bar, 50 μm) and statistical analysis of NLRP3 in the liver of *Tet2*^WT^-CCl_4_ and *Tet2*^ΔMye^-CCl_4_ mice (*n* = 5 for each group). Red: anti-NLRP3; blue: DAPI. **(I)** IHC staining (scale bar, 50 μm) and statistical analysis of NLRP3 in the liver of *Tet2*^WT^-CCl_4_ and *Tet2*^ΔMye^-CCl_4_ mice (*n* = 5 for each group). **(J)** Inhibition of IL-1β in the serum of oil- or CCl_4_-treated *Tet2*^WT^ and *Tet2*^ΔMye^ mice (*n* = 5 for each group). **(K and L)** Change of Ccl2 (K) and Ccl8 (L) in the serum of oil- or CCl_4_-treated *Tet2*^WT^ and *Tet2*^ΔMye^ mice after anti–IL-1β Ab treatment (*n* = 4–5 for each group). **(M and N)** Effect of anti–IL-1β Ab treatment on the mRNA levels of *Acta2* and *Col1a1* (*n* = 5 for each group). **(O)** Picrosirius red staining of collagen deposition in the liver tissue of *Tet2*^WT^-CCl_4_ and *Tet2*^ΔMye^-CCl_4_ mice after treatment of anti–IL-1β Ab (*n* = 5 for each group). Data are representative of at least two independent experiments with similar results (B, C, and E–O). All data are shown as mean ± SD and were analyzed by two-tailed, paired Student’s *t* test (B, C, and H, and I) or two-way ANOVA with Sidak’s multiple comparison test (E–G and J–N). ***P < 0.001; **P < 0.01; *P < 0.05; P > 0.05 not significant (ns).

These findings were validated in a bile duct ligation (BDL)-induced fibrosis model (Fig. S5 A). Old-BMT mice exhibited increased intrahepatic MDMs infiltration (Fig. S5 B); elevated serum Ccl2, Ccl8, and IL-6 levels (Fig. S5 C); collagen deposition (Fig. S5, D and E); Col IV (Fig. S5 F); and HA levels (Fig. S5 G) in comparison with young-BMT mice. Bindarit plus IL-6 Abs treatment significantly mitigated fibrosis-related indexes in both young-BMT and old-BMT mice (Fig. S5, D–G). In conclusion, *Tet2*^ΔMye^-induced myeloid hematopoiesis exacerbates liver fibrosis in both young and aging mice through a consistent Ccl2/Ccl8–pMDMs–IL-6–HSCs pathway highlighting a unified therapeutic target for age-associated fibrosis.

### *Tet2*-mutant clonal hematopoiesis (CHIP) in aged individuals is associated with severe liver fibrosis and elevated Ccl2/Ccl8 levels

**Figure 10.**
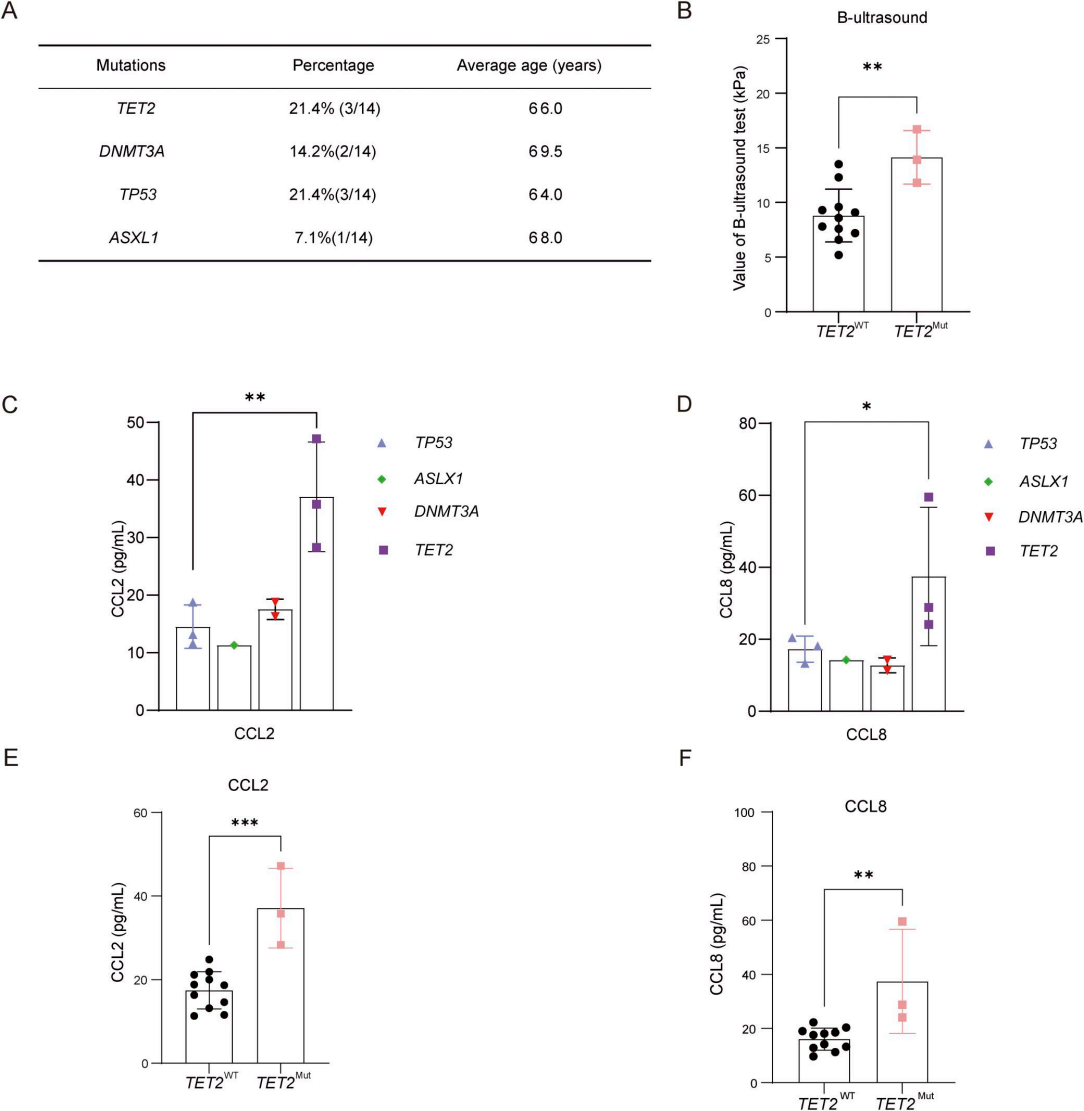
***TET2*** ^**Mut**^ **-related CHIP in aged individuals is associated with severe liver fibrosis and elevated Ccl2/Ccl8 levels. (A)** The mutation frequency of genes known to be drivers of clonal hematopoiesis in aged individuals. The top four most frequently mutated genes in CHIP for the current study population were as follows: *DNMT3A*, *TET2*, *TP53*, and *ASXL1*. **(B)** The severity of liver fibrosis in *TET2*^WT^ and *TET2*^Mut^ population with liver fibrosis.* TET2*^WT^, *n* = 11; *TET2*^Mut^, *n* = 3. **(C and D)** The different levels of Ccl2 (C) and Ccl8 (D) in peripheral blood of patients with *DNMT3A*, *TET2*,* TP53*, and *ASXL1 *mutations. *TP53*^Mut^, *n* = 3; *ASLX1*^Mut^, *n* = 1; *DNMT3A*^Mut^, *n* = 2; *TET2*^Mut^, *n* = 3. **(E and F)** The levels of Ccl2 (E) and Ccl8 (F) in peripheral blood of *TET2*^WT^ and *TET2*^Mut^ population with liver fibrosis. TET2^WT^, *n* = 11; TET2^Mut^, *n* = 3. Data are the accumulative results from at least two independent experiments (A–F). All data are shown as mean ± SD and were analyzed by two-tailed, unpaired Student’s *t* test (B, E, and F) or two-way ANOVA with Sidak’s multiple comparison test (C and D). ***P < 0.001; **P < 0.01; *P < 0.05; P > 0.05 not significant (ns).

To investigate whether *Tet2*-mutant CHIP in leukocytes contributes to accelerated liver fibrosis and altered chemokine signaling, we performed whole-exome sequencing (WES) on peripheral blood samples from 14 patients with liver fibrosis quantitatively assessed by ultrasound elastography to identify CHIP-associated mutations. Consistent with prior reports, we detected recurrent somatic mutations in DNA methyltransferase 3 alpha (*DNMT3A*), *TET2*, tumor protein p53 (*TP53*), and ASXL transcriptional regulator 1 (*ASXL1*). Notably, *TET2* mutations were present in 3 of 14 (21.4%) patients with liver fibrosis (Fig. 10 A).

In our cohort of 14 patients with confirmed liver fibrosis, targeted analysis of CHIP-associated driver mutations in peripheral blood leukocytes revealed the following distribution of canonical age-related variants: *TRP53* mutations were detected in three cases (21.4%), *ASXL1* in one case (7.1%), *DNMT3A* in two cases (14.3%), and *TET2* in three cases (21.4%). Notably, one patient demonstrated concurrent *TP53* and *ASXL1* mutations. Furthermore, individuals with *TET2*^Mut^ CHIP exhibited significantly more advanced liver fibrosis and relatively higher levels of Ccl2 and Ccl8 in serum compared with *TET2* WT (*TET2*^WT^) counterparts, even after adjusting for co-occurring mutations (Fig. 10, B–D). Plasma analysis revealed markedly elevated levels of the pro-inflammatory chemokines Ccl2 and Ccl8 in TET2^Mut^ carriers (Fig. 10, E and F).These findings mirror our experimental findings in murine models and demonstrate that *TET2*^Mut^-related CHIP in aging individual promotes liver fibrogenesis and is linked to a distinct inflammatory signature characterized by Ccl2/Ccl8 upregulation.

## Discussion

The role of *Tet2* in regulating chronic inflammation and myeloid cell function has been extensively studied, with prior research highlighting its importance in suppressing inflammatory responses in monocytes and macrophages (Cull et al., 2017; Lv et al., 2018; Zhang et al., 2015; Ichiyama et al., 2015). *Tet2* deficiency in macrophages disrupts the resolution of inflammation, a finding relevant to the immune environment in *Tet2*-mutant clonal hematopoiesis and myeloid cancers (Cull et al., 2017). However, the specific impact of *Tet2* deficiency on liver fibrosis, particularly in the context of aging and myeloid hematopoiesis, remains poorly understood. Our study provides novel insights into how myeloid-specific *Tet2* deficiency exacerbates liver fibrosis through the Ccl2/Ccl8–pMDMs–IL-6–HSCs axis, offering a potential therapeutic avenue for targeting fibrosis in populations with abundant aging individuals with *TET2* mutations.

Chemokines Ccl2 and Ccl8 are well-known mediators of monocyte and macrophage recruitment, acting through receptors Ccr1, Ccr2, and Ccr3 (Townson and Liptak, 2003). Our findings reveal that *Tet2*^−/−^ pMDMs are a major source of elevated Ccl2 and Ccl8 in *Tet2*^ΔMye^-CCl_4_ mice, and *Tet2*^−/−^ monocytes showed an increased expression of Ccr2 and Ccr3, which, thus, created a positive feedback loop that amplifies MDMs infiltration and liver fibrosis. This aligns with previous studies demonstrating the central role of chemokine signaling in chronic liver diseases (Galastri et al., 2012; Tacke, 2018; van der Heide et al., 2019). However, our work uniquely identifies *Tet2*^−/−^ pMDMs as a critical driver of this process, indicating that targeting these cells could disrupt the fibrotic cascade. The efficacy of Bindarit in reducing fibrosis further supports the therapeutic potential of chemokine inhibition, though its long-term effects and broader applicability require further investigation. The failure of cenicriviroc, a dual CCR2/5 inhibitor, in recent phase 3 clinical trials for metabolic dysfunction–associated steatohepatitis fibrosis (Anstee et al., 2024) highlights the complex challenges in translating chemokine-targeted therapies into clinical efficacy. While our preclinical study demonstrates that Bindarit-mediated *Ccl2*/*Ccl8* inhibition attenuates liver fibrosis in *Tet2*^ΔMye^-CCl_4_ mice, the discordance with cenicriviroc trial outcomes underscores the challenges of clinical application of our findings. Several factors may account for this disparity. First, human fibrotic liver diseases exhibit substantial etiological and molecular heterogeneity, suggesting that interventions targeting chemokine networks may require selection of patients with specific pathogenic drivers—such as individuals with *TET2*-mutant clonal hematopoiesis, an aged population highlighted in our study. Second, our data indicate that the fibrogenic mechanism downstream of *Tet2* deficiency involves both Ccl2/Ccl8 and Il-6 signaling; thus, dual blockade of both axes may be necessary for maximal therapeutic effect, potentially explaining the limited efficacy of cenicriviroc monotherapy. Third, elevated levels of CCL2 and CCL8 were detected in the serum of elderly liver fibrosis patients carrying TET2 mutations, supporting the clinical relevance of a *TET2*–*CCL2/8* regulatory axis in human disease, which provide a mechanistic basis for the potential clinical application of Bindarit. Moreover, Bindarit has already been evaluated in clinical trials for proliferative lupus nephritis and coronary stent restenosis, where it demonstrated a favorable safety profile. Finally, Il-6 inhibitors and monoclonal Abs are relatively well established in clinical use. Together, these aspects provide strong support for the translational feasibility of our proposed therapeutic strategy.

The discrepancy between our findings and those by Pandey et al. (2022), who reported no increased susceptibility to CCl_4_-induced fibrosis in hematopoietic *Tet2*-deficient mice, underscores the importance of model-specific factors. Differences in mouse strains, fibrosis induction methods, and the specificity of *Tet2* deletion (systemic vs. hematopoietic) may account for these contrasting results. Our use of myeloid-specific *Tet2* KO mice provides a more precise understanding of the role of *Tet2* in myeloid cells, highlighting their contribution to fibrosis progression. This emphasizes the need for standardized models to reconcile divergent findings in the field.

*Il-6* is a well-established mediator of inflammation and fibrosis, with prior studies linking its upregulation to signal transducer and activator of transcription 3 (STAT3) activation and HSCs trans-differentiation (Xiang et al., 2018; Choi et al., 2019; Kagan et al., 2017). Previous studies demonstrated TET2’s transcriptional regulation of IL-6 in cultured macrophages by microRNA (Jiang et al., 2019). Our findings extend this understanding by identifying *Tet2*^−/−^ pMDMs as a key source of IL-6 in liver fibrosis. Interestingly, we observed no significant differences in NLRP3 expression between *Tet2*^WT^-CCl_4_ and *Tet2*^ΔMye^-CCl_4_ mice (Fig. S5, H and I), contrasting with Wong et al.’s findings in a steatohepatitis model (Wong et al., 2023). To definitively exclude the regulatory role of the IL-1β–NLRP3 inflammatory axis in our liver fibrosis model (McClatchy et al., 2023), we administered an IL-1β–neutralizing Ab to CCl_4_-induced fibrotic mice. While IL-1β blockade effectively reduced serum IL-1β levels (Fig. S5 J), it failed to significantly alter circulating Ccl2 and Ccl8 concentrations in either *Tet2*^WT^ or Tet2^ΔMye^ mice (Fig. S5, K and L). Hepatic fibrosis markers (*Acta2* and *Col1a1*) and collagen deposition remained elevated in *Tet2*^ΔMye^-CCl_4_ mice despite IL-1β neutralization (Fig. S5, M–O). These data conclusively demonstrated that IL-1β–independent mechanisms drive *Tet2* deficiency–aggravated liver fibrogenesis. This discrepancy may reflect differences in disease etiology and immune responses, highlighting the complexity of *Il-6* signaling in liver fibrosis.

Our study reveals a novel mechanism by which *Tet2* regulates the stability of *Ccl2* and *Ccl8* mRNAs through 5mC-to-5hmC oxidation in ARE motifs, modulating the binding of *Ybx1*, *Elavl1*, and *Zfp36*. This expands the growing understanding of *Tet2* as a multifaceted regulator of posttranscriptional gene expression. Notably, our findings align with and complement the work of He et al. (2023), who demonstrated that *Tet2* suppresses mechanistic target of rapamycin complex 1 (mTORC1) signaling by destabilizing urea cycle enzyme mRNAs through 5mC oxidation, thereby limiting arginine production and cell growth (He et al., 2023). While both studies highlight *Tet2*’s role as an RNA epitranscriptomic modulator, they reveal distinct yet interconnected biological outcomes. While He et al. demonstrated *Tet2*’s regulation of urea cycle enzymes (*Asl* and *Ass1*) linking RNA methylation to metabolic reprogramming, our study for the first time demonstrates *Tet2*’s novel role in stabilizing chemokine mRNAs in pMDMs, connecting RNA methylation to inflammatory responses, suggesting *Tet2* exhibits target specificity for distinct mRNA motifs to differentially regulate cellular processes. Both studies show *Tet2*-mediated 5hmC disrupts *Ybx1* and *Elavl1* binding but with opposing functional consequence-promoting decay of metabolic mRNAs in hepatocytes while stabilizing inflammatory mRNAs in macrophages, a divergence potentially explained by cell type–specific contexts or our newly identified involvement of *Zfp36* in antagonizing *Ybx1* and *Elavl1* for ARE-mediated decay. Beyond He et al.’s proposed mTORC1 inhibition strategy for *Tet2*-deficient tumors, our findings extend therapeutic potential to inflammation-driven pathologies by targeting the *Tet2*-*Ybx1*/*Elavl1*-*Zfp36* axis, particularly in diseases where *Ccl2* and *Ccl8* play key roles, thereby establishing *Tet2* as a versatile regulator capable of coordinating both metabolic and immune responses through distinct but mechanistically related pathways.

Our observation of increased CD45.2^+^ki67^+^F4/80^+^ cells in recipient mice transplanted with *Tet2*^−/−^ myeloid cells indicates that *Tet2* deficiency confers a survival and proliferative advantage to myeloid cells in inflammatory microenvironments. This finding aligns with previous reports of *Tet2*’s role in regulating myeloid cell differentiation and expansion (Wong et al., 2023; Meisel et al., 2018). However, our study uniquely demonstrates that *Tet2*^−/−^ MDMs exhibit enhanced proliferation and chemokine production, driving fibrosis progression. This expands the current understanding of *Tet2*’s role in myeloid hematopoiesis and highlights its potential as a therapeutic target in liver fibrosis.

The limited efficacy of IL-6 neutralization alone in our study indicates that persistent MDMs infiltration and IL-6 production may undermine monotherapy approaches. In contrast, the combination of Bindarit and IL-6 Abs significantly enhanced anti-fibrotic effects, highlighting the importance of targeting both chemokine-driven recruitment and IL-6–mediated HSCs activation. This aligns with emerging trends in fibrosis therapy, which emphasize multi-target approaches to address the complex interplay of inflammatory and fibrotic pathways. However, the translational potential of this strategy requires further validation in preclinical and clinical studies.

Although our study provides compelling evidence for the role of *Tet2*^−/−^ pMDMs in liver fibrosis, several questions remain unresolved. For instance, the precise mechanisms through which *Tet2* deficiency enhances Il-6 production and HSCs activation are unclear. It is worth noting that several previous studies—including those by Zhang et al. (2015), Cull et al. (2017), and Jiang et al. (2019)—have indeed documented a role for *Tet2* in modulating Il-6 expression in immune cells. These works collectively demonstrate that *Tet2* can either promote or suppress Il-6 depending on cellular context and mechanism, involving DNA hydroxymethylation, microRNA-metabolite cross talk, or recruitment of histone deacetylases. Given that the cell-intrinsic mechanisms of *Tet2* in macrophage *Il-6* regulation have been extensively explored in prior *in vitro* studies, our work instead focused on the novel *in vivo* axis of monocyte recruitment and pMDMs expansion driven by *Tet2* deficiency, which represents the core finding of our research. Beyond this, previous reports have implicated STAT3, MAPK, and JAK/STAT pathways in Il-6–mediated fibrosis; however, whether these pathways are similarly activated in *Tet2*^−/−^ pMDMs requires further investigation. In addition, the impact of *Tet2* deficiency on other immune cell populations, such as T cells and neutrophils, requires exploration, as these cells may also contribute to fibrosis progression.

Our study establishes that myeloid-specific *Tet2* deficiency exacerbates liver fibrosis through the Ccl2/Ccl8–pMDMs–IL-6–HSCs axis, with *Tet2*^−/−^ pMDMs driving chemokine production, MDMs infiltration, and HSCs activation. These findings highlight the potential of combination therapies targeting MDMs recruitment and *Il-6* signaling as a novel approach to fibrosis treatment, particularly in populations with abundant aging individuals with *TET2* mutations.

## Materials and methods

### Mice

C57BL/6/J, Alb-cre (B6.Cg-Speer6-ps1Tg(Alb-cre)21Mgn/J) and C57BL/6/J, and LysM-Cre (B6. Lyz2tm1(cre)Ifo)) were purchased from the Jackson Laboratory. The systemic *Tet2* KO mice were kindly provided by Guoliang Xu, the Chinese Academy of Sciences, Beijing, China. C57BL/6J, CD45.1(B6.SJL-Ptprca Pepcb/BoyJ), and *Tet2*^flox/flox^ ((B6;129S-*Tet2*^tm1^.1Iaai/J)) mice were purchased from Southern Model Animal Center (Shanghai, China). To generate Alb-specific and LysM-specific *Tet2*-KO mice, *Tet2*^flox/flox^ mice were crossed with Alb-cre and Lyz2-cre mice. All mice are housed and maintained, five mice per cage, in the animal SPF facility of Fudan University (Shanghai, China). Systemic *Tet2* KO (Sys) mice, Alb-specific *Tet2* KO (Alb) mice, and Lyz2 cell-specific *Tet2* KO (Mye) mice, along with WT littermate controls, were obtained for all experiments in this study. All animal procedures were conducted by the Guidelines for the Care and Use of Laboratory Animals and were approved by the Ethics Committee of the Institute of Fudan University.

### Human samples

Patients diagnosed with liver fibrosis were recruited from Zhongshan Hospital, Fudan University (Shanghai, China), according to the following inclusion criteria: (1) age ≥18 years; and (2) confirmed diagnosis of liver fibrosis. Peripheral blood samples were collected from all participants. Written informed consent was obtained from each patient prior to enrollment. The study (B2024-247) was conducted in full compliance with international ethical standards and was approved by the Institutional Ethics Committee of Zhongshan Hospital, Fudan University, Shanghai, China.

### Reagents

Bindarit (AF283; MCE) were purchased from MedChemExpress. Clodronate Liposomes or Clodronate PBS (40337ES08) was purchased from Yeasen (Shanghai, China). InVivo Mab anti-mouse Ccr2 (BE0457), Ccr3 (BE0316), IL-6 (BE0046), and IL-1β (BE0246) were purchased from Bio-X-cell.

### Cell cultures

THP1 cell line was purchased from ATCC and was cultured in high-glucose DMEM (Gibco) supplemented with 10% FBS (Gibco) and 1% penicillin–streptomycin (P/S, 100 U/ml; Hycone). All cell lines were maintained at 37°C in a humidified incubator with 5% CO_2_. For MDMs culture, the newly isolated MDMs from the liver were seeded in Roswell Park Memorial Institute (RPMI) 1640 containing 5% FBS and 1% P/S and were incubated at 37°C, in a humidified incubator with 5% CO_2_. For HSCs isolation and culture, the liver tissues were prepared for HSCs separation using gradient Percoll solutions by density-gradient centrifugation as established previously. HSCs were cultured in DMEM with 10% FBS (Sigma-Aldrich), 20 mM HEPES, 100 μg/ml streptomycin, and 100 U/ml penicillin. As for the co-culture of MDMs with HSCs, we used a Transwell (Corning incorporated) system. 5 × 10^5^ MDMs were seeded in the lower chamber, and 5 × 10^5^ HSCs were placed in the top champers with a 0.4-μm pore size. After 48 h, the supernatant, MDMs, and HSCs were collected and processed for further analysis.

### CRISPR/Cas9-mediated *Tet2* KO and knock-in of a catalytic mutant allele

For *Tet2* KO cell line construction, single-guide RNAs (sgRNAs) were designed with CRISPR tool and then cloned into lentiCRISPR v2 vector (Table S2). Targeting vector and single-stranded DNA donor were co-transfected in cells for 24 h and followed by 2 days of puromycin selection. For the catalytic mutant cell line construction, a sgRNA was designed near the catalytic domain to enhance the efficiency of homology-directed repair. A single-strand oligonucleotide donor template, encoding a well-characterized catalytic-dead point mutation, was co-delivered. Lentiviral supernatants were packaged with psPAX2 and pMD2.G plasmid. Target cells were incubated with the viral supernatant supplemented with 2 µg/ml polybrene for 24 h. All lentiviral work was conducted using biosafety level 2

### Mouse genotyping

For genotyping, sterile scissors were used to cut 0.5-mm tail tissue, and genomic DNA was extracted using a genome extraction kit. PCR was performed with a standard thermocycler and blue EasyTaq enzyme (Vazyme). Reaction conditions varied by genotype: *Tet2*^−/−^: 94°C 5 min → 35 cycles (94°C 30 s, 57.5°C 30 s, and 72°C 30 s) → 72°C 5 min; *Tet2*^f/f^: 94°C 3 min → 35 cycles (94°C 30 s, 60°C 30 s, and 72°C 45 s) → 72°C 5 min; *Tet2*^Alb^: 94°C 4 min → 35 cycles (94°C 30 s, 60°C 30 s, and 72°C 30 s) → 72°C 5 min; and *Tet2*^Mye^: 94°C 3 min → 35 cycles (94°C 30 s, 68°C 30 s, and 72°C 30 s) → 72°C 5 min; 10 µl of PCR products were resolved on 1.5% agarose gels, and genotypes were determined by band pattern (Table S2).

### Mouse model and treatments

To construct a hepatic fibrosis mouse model, olive oil or 20% (vol/vol) CCl_4_ solution (prepared with olive oil) were intraperitoneally injected into the control and modeling groups, respectively, at a volume of 5 μl/gram of mouse body weight, twice weekly for 7–8 wk. The mice used to establish the liver fibrosis model were 7-wk-old males housed in a specific pathogen-free environment with a 12-h light/12-h dark cycle. For the BDL mouse model, liver fibrosis was induced by bile ductal ligation surgery according to previously published procedures. Briefly, mice were anesthetized via intraperitoneal injection of tribromoethanol and subjected to abdominal surgery. The liver was gently elevated using a cotton swab to expose the bile duct. The duct was then carefully dissociated from the adjacent portal vein and hepatic artery, double ligated with sutures, and finally transected between the two ligatures.

### Myeloid cell isolation and transplantation

7-wk-old male CD45.2 *Tet2*^+/+^and CD45.2 *Tet2*^−/−^ mice were euthanized, and spleens were harvested. Single-cell suspensions were generated using mechanical dissociation (5-ml syringe piston) and filtered through 70-μm strainers. CD45.2^+^CD11b^+^ myeloid cells were sorted by flow cytometry (BD Fortessa), with purity confirmed by post-sort analysis. To optimize engraftment, 8 × 10^6^ unfractionated CD11b^+^ cells (split into 4 daily intravenous doses of 2 × 10^6^ cells) were transferred into CD45.1^+^ recipients. 3 wk after the final transplantation, mice were administrated with CCl_4_ solution to established liver fibrosis model (2 μl/g, twice weekly for 6 wk). Fibrosis severity was evaluated by Sirius red/Col IV staining, HA quantification, and liver function tests. Intrahepatic CD45.2^+^CD11b^+^ cells were analyzed by flow cytometry, IHC, and IF; peripheral blood engraftment was assessed weekly by flow cytometry.

### *In vivo* inhibitors treatment

To establish a model of hepatic fibrosis, age-matched *Tet2*^WT^ and *Tet2*^ΔMye^ littermate mice were subjected to intraperitoneal injections of CCl_4_. Beginning 1 wk after the initiation of CCl_4_ treatment, mice received either Bindarit (AF283; MedChemExpress) dissolved in PBS or PBS alone as a vehicle control. Bindarit was administered via oral gavage at a dosage of 50 mg/kg body weight, once per week for a total of seven doses. For MDMs depletion, Clodronate liposomes (Yeasen) or PBS-loaded liposomes (control) were injected intravenously at a volume of 150 μl per mouse on a weekly basis, also for seven doses, starting 1 wk after the commencement of CCl_4_ administration. For cytokine and chemokine receptor neutralization experiments, monoclonal Abs against IL-6 (BE0046; Bio X Cell), IL-1β (BE0246; Bio X Cell), Ccr2 (BE0457; Bio X Cell), and Ccr3 (BE0316; Bio X Cell) were used. Abs were administered intraperitoneally at a dose of 200 μg per mouse every 2 days for a total of eight injections. Ab treatment was initiated 1 wk after the start of CCl_4_ exposure. Hepatic fibrosis was assessed through multiple endpoints: collagen deposition was evaluated via Sirius red staining and IHC detection of collagen type I; serum HA levels were measured by ELISA; and hepatic injury was assessed by quantifying serum activities of ALT and AST using standard biochemical assays.

### Isolation of liver-infiltrating MDMs

In CD45.1 chimeric mice or *Tet2*^ΔMye^ mice with liver fibrosis, 2 g of fresh liver tissue was collected after the mice were sacrificed. The liver tissue was minced into pieces with sterile scissors. Then, the minced liver tissue was digested with 10 ml of digestion solution containing Col IV (200 U/ml) and pronase (50 U/ml) in a 37°C water bath for 1 h. After the liver tissue was digested into flocculent residues, the digestion suspension was filtered through a 70-μm strainer (BD) to obtain a single-cell suspension of the liver tissue. The filtered solution was centrifuged at 350 *g* for 10 min at 4°C and washed twice with pre-chilled PBS. MDMs, defined as CD45.2^+^CD11b^+^ F4/80^+^Ly6c^high^, were obtained by a flow cytometry sorter (BD FACS Melody). The purity of sorted cells was confirmed by a flow cytometer (BD, FORTESSA).

### Expansion assay of MDMs *in vitro*

*Tet2* ^+/+^pMDMs and *Tet2*^−/−^ pMDMs were collected and confirmed by flow cytometry. *Tet2*^+/+^pMDMs and *Tet2*^−/−^ pMDMs were labeled with 2 μM CFSE (Sigma-Aldrich) in PBS for 10 min at 37°C in the dark. Labeled MDMs were washed four times with PBS and centrifuged at 350 *g* to harvest cells. The CFSE-labeled MDMs (2 × 10^4^ cells/well) were plated in RPMI 1640 containing 5% FBS and 1% P/S and were incubated in a 37°C, 5% CO_2_ humidified incubator. After 4 days, cells were harvested, and CFSE signals were measured by flow cytometry.

### Co-culture of MDMs and HSCs

For the co-culture of MDMs and HSCs, *Tet2*^+/+^and *Tet2*^−/−^ MDMs were isolated from CCl_4_-treated *Tet2*^WT^ and *Tet2*^ΔMye^ mice, respectively, and *Tet2*^+/+^and *Tet2*^−/−^ HSCs were isolated from oil-treated *Tet2*^WT^ and *Tet2*^ΔMye^ mice, respectively. The isolated MDMs and HSCs were resuspended to a concentration of 3 × 10^6^/ml with high-glucose DMEM containing 10% FBS (Sigma-Aldrich), 20 mM HEPES, 100 μg/ml streptomycin, and 100 IU/ml penicillin. Noncontact co-culture of MDMs and HSCs was performed in 12-well transwell chambers. Totally, 5 × 10^5^ HSCs were seeded into the top chamber, and 5 × 10^5^ MDMs were seeded into the lower chamber of the transwell plate. The co-cultures were maintained at 37°C in an atmosphere of 5% CO_2_ for 48 h. Then, the supernatant was collected, and the levels of IL-6 and Ccl8 were measured by ELISA. HSCs were collected, and the mRNA and protein of α-SMA and collagen I were detected using western blotting and RT-PCR.

### Construction of chimeric-aged mice mimicking *Tet2*^−/−^-related clonal hematopoiesis

Unfractionated BMCs were isolated from femurs and tibias of 8-wk-old unfractionated CD45.2 *Tet2*^ΔMye^ female mice as reported previously. Briefly, BMCs were flushed from both ends of the bone shafts into a 15-ml Falcon tube fitted with a 70-μm filter using a 25-G needle attached to a 10-ml syringe containing DMEM supplemented with 5% (vol/vol) FBS and 2 mmol/l EDTA. Then, 0.5 × 10^6^ CD45.2 *Tet2*^ΔMye^ BMCs (20%) and 2 × 10^6^ CD45.1 *Tet2*^+/+^BMCs (80%) were resuspended in 200 μl sterile PBS and injected into the tail veins of the x-ray irradiated (9.5 Gy) 7-wk-old (young-BMT) and 18-mo-old (old-BMT) CD45.1 WT recipient mice. CCl_4_ was administrated for 6 wk to construct liver fibrosis mouse model post-BMT procedure.

### Picrosirius red staining

The mice were anesthetized, and the livers were isolated and fixed with 4% paraformaldehyde (PFA) overnight. Tissue was paraffin embedded and cut into 5-μm paraffin sections. Sections were incubated at 60°C for 2 h and dewaxed by xylene. Sections were hydrated with 100%, 95%, 85%, 75%, or 50%, ddH_2_O, 5 min for each incubation. The hydrated sections were treated with celestite blue solution for 10 min and washed thrice with ddH_2_O, 5 min each time. Sirius staining solution and saturated picric acid solution were added dropwise and treated at room temperature for 20 min. Ethanol containing 1% hydrochloric acid was used for differentiation, and gradient ethanol was used for dehydration. Sections were sealed with neutral resin after clarification using thrice xylene solution incubation. The coverslip was fixed with nail polish. Finally, sections were observed and photographed under a microscope.

### Immunohistochemistry staining

Liver was taken out and fixed in 4% PFA overnight. The fixed liver tissue was embedded in paraffin wax and cut into 5-μm sections. IHC were conducted as previously established protocol. Briefly, tissue sections were deparaffinized thrice in xylene and rehydrated through an ethanol gradient. For IHC, boiling, pH 9.0, EDTA buffer was used for antigen retrieval, and tissues on sections were circled with oil marker pen and incubated in a 0.3% H_2_O_2_ solution diluted by methanol to remove peroxidase in liver tissue. After liver tissue was blocked in normal goat serum or 3% BSA according to primary Ab instructions, samples were incubated with rat monoclonal anti-F4/80 (1:100, 29414-1-AP; Proteintech), rabbit monoclonal anti-Ccl8 (1:400, 20049-1-AP; Proteintech), rabbit polyclonal to Ccl2 (1:200, 26161-1-AP; Proteintech), anti-α-SMA (1:200, ab124964; Abcam), anti-collagen Ⅰ (1:200, 131984; Absin), and anti-CD45.1 (1:200, 84325-2-RR; Proteintech) for 12 h at 4°C, followed by 1 h incubation with an instant biotinylated rabbit anti-mouse lgG Ab. Sections were incubated with SABC for 30 min at 37°C, followed by 3,3′-diaminobenzidine and hematoxylin staining. Finally, sections were sealed with neutral resin after clarification through triple xylene solution incubation. All sections were scanned under a microscope.

### IF staining

Paraffin-embedded tissue sections (4 μm) were deparaffinized in xylene and rehydrated through a graded ethanol series. Antigen retrieval was performed using EDTA buffer (pH 9.0) under microwave heating. After cooling and washing in PBS, sections were blocked with 3% BSA for 30 min at room temperature. Primary Abs against CD45.2 (60287-1-Ig; Proteintech), F4/80 (ab300421; Abcam), Ly6c (65296-1-Ig; Proteintech), CD68 (ab53444; Abcam), CD206 (ab300621; Abcam), Inos (22226-1-AP; Proteintech), and NLRP3 (22226-1-AP; Proteintech) were applied and incubated overnight at 4°C. Following PBS washes, fluorescently conjugated secondary Abs (fluorescein isothiocyanate, PE, Cy3) were incubated for 1 h min at room temperature in the dark. Nuclei were counterstained with DAPI (ab104139; Abcam), and slides were mounted with anti-fade medium. MDMs were identified as CD11b^+^F4/80^+^Ly6c^high^ cells; pMDMs were defined as CD68^+^ MDMs; activated MDMs were further specified as inducible nitric oxide synthase (iNOS^+^) MDMs. Images were scanned using a 3DHISTECH panoramic fluorescence slide scanner (model Pannoramic MIDi) and subjected for quantification of positive staining with Lab Image Pro V6.0 software.

### Measurement of liver function

The fresh peripheral blood was collected and placed at room temperature for 2 h. Serum was isolated from peripheral blood by centrifuging 3,500 rpm for 15 min at 25°C. Liver function indexes, including ALT and AST, were measured by SimuMu Biotechnology, Shanghai, China. ALT and AST were, respectively, measured by alanine aminotransferase assay kit (#S03030; Leidu) and aspartate aminotransferase assay kit (#S03040; Leidu) following the manufacturer’s instructions.

### Samples preparation and ELISA analysis of cytokines

ELISA was used to detect the levels of Col IV (#20024; Ruixin Biotechnology), HA (#20067; Ruixin Biotechnology), Ccl8 (#RX27820; Ruixin Biotechnology), Ccl2 (MG9180; Fantia), TNFα (#KE10002; Proteintech), IL-2 (#BGM4904; Bangjing), IL-1α (KE10098; Proteintech), IL-1β (KE10003; Proteintech), and IL-6 (#KE10091; Proteintech) in serum and liver tissue grinding fluid. For liver tissue sample, 0.2 g of liver tissue was grinded into fine powder in liquid nitrogen with a mortar, and 200 μl radioimmunoprecipitation assay (RIPA) lysis buffer containing protease inhibitors was added and further homogenized it with an electric grinder. The homogenate was filtered by 70-μm strainer, and the percolate was centrifuged at 12,000 *g* at 4°C for 15 min. The supernatant was carefully collected for ELISA analysis. For serum samples, 400 μl of blood was collected from mouse orbit and placed at room temperature for 2 h. The blood was centrifuged at 3,500 rpm for 15 min at room temperature, and the upper serum was carefully collected. The procedure for ELISA was performed as the instructions of manufacturer. Briefly, standard curve was produced, and the blank well, standard well, and sample wells were set. Samples were incubated with horseradish peroxidase and were washed repeatedly with washing solution five times. After adding substrate to each well, the reaction was incubated at room temperature for 15 min and stopped with stop solution. The absorbance value of each well was measured at a wavelength of 450 nm within 15 min. The concentration of samples was calculated using a standard curve.

### Western blotting

Cells or liver tissue lysed by liquid nitrogen were resuspended and lysed by ice-chilled RIPA lysis buffer containing protease inhibitor cocktail (Millipore) for 1 h at 4°C. Protein concentration was quantified using a bicinchoninic acid kit, and Lamin B or Gapdh was used as the loading control. Western blotting was performed as established protocols. Briefly, 10 μg of protein was processed using 10% SDS-PAGE. Proteins in SDS-PAGE gel was transferred to nitrocellulose membrane for 1.5 h. The targeted strips were cut according to molecular weight and incubated by 5% skim milk at room temperature for 1 h. After washing thrice with Tris-buffered saline with Tween washing, targeted strips were incubated with Collagen I (1:1,000, 131984; Absin), α-SMA (1:2,000, ab124964; Abcam), Ybx1 (1:2,000, 20339-1-AP; Proteintech), Zfp36 (1:1,000, 12737-1-AP; Proteintech), Elavl1 (1:2,000, 11910-1-AP; Proteintech), and Lamin B (1:5,000, 66095-1; Proteintech) primary Ab at 4°C overnight and then incubated with secondary Ab (anti-mouse IgG [1:10,000, 6229; SAB]) and anti-Rabbit IgG (1: 10,000, 8715; SAB). Finally, target bands were developed by Smart-Enhanced Chemiluminescence solution, and protein expression was quantified using ImageJ software.

### RNA extraction and RT-qPCR

Total RNA was extracted from liver tissue, peritoneal macrophage, and cell lines according to established protocol. The concentration of RNA was measured by using a NanoDrop 2000 (Thermo Fisher Scientific). 1 μg RNA was used for cDNA synthesis using the Prime Script RT reagent Kit with gDNA Eraser (RR047Q; Takara), and cDNA product was used as template after fivefold dilution with ddH_2_O. Gene expression level was measured by SYBR (Roche) intensity in 384-well plate on ABI Prism 7500 (Applied Biosystems). The expression level of target genes was normalized to *Gapdh* using the ΔC(t) method. All primers used in this study were listed in Table S2.

### Flow cytometry analysis

For tissue, 0.5 g fresh liver of mice was minced into pieces with sterile scissors and digested into single-cell suspensions by Col IV (200 U/ml) and Pronase (50 U/ml) for 3.5 h at 37°C water bath. The single-cell suspension was filtered using a 70-μm cell strainer (BD) and washed with pre-chilled PBS (1% FBS in PBS). For peripheral blood, 100 μl peripheral blood was collected into EP tube with 0.5 mM EDTA, and the red blood cells were removed with 1 ml lysing buffer (cat. 555899; BD). Then, cells were collected by centrifuging at 350 *g* for 5 min. For staining: cell pellet prepared from tissue or peripheral blood was resuspended in 40 μl staining buffer. Samples were blocked with 2 μl Fc block for 10 min at 4°C before staining with targeted fluorophore-conjugated Abs. Single-cell resuspension (1 × 10^6^) and 5 μl specific fluorophore-conjugated Abs were thoroughly mixed and incubated for 30 min on ice in darkness. Finally, samples were washed twice with pre-chilled PBS and suspended in FACS buffer for analysis by LSR Fortessa (BD Biosciences). All files were analyzed by Flow Jo software (FlowJo V10). The fluorescence-conjugated Abs used in the study were listed in Table S3.

### RIP-qPCR

RIP assay was performed to investigate the binding of Elavl1, Zfp36, and Ybx1 to *Ccl2* and *Ccl8* mRNAs in bone marrow–derived pMDMs. BMCs isolated from *Tet2* WT and *Tet2* KO mice were differentiated into pMDMs by culturing in RPMI-1640 medium supplemented with 10% FBS and 20 ng/ml macrophage CSF for 7 days, with medium replacement every 48 h. To induce pro-inflammatory polarization, the differentiated macrophages were then stimulated with 100 ng/ml LPS and 20 ng/ml IFN-γ for 24 h. Macrophage differentiation and polarization were confirmed by flow cytometry (CD11b^+^F4/80^+^ >90%). The prepared pMDMs were cross-linked with 1% formaldehyde for 10 min at room temperature; quenched with 125 mM glycine; and lysed in buffer containing 1% SDS, 10 mM EDTA, and 50 mM Tris-HCl (pH = 8.1) with protease/RNase inhibitors. Chromatin was sheared by sonication (15 cycles of 30 s ON/OFF) and precleared with protein A/G magnetic beads. For each RIP, 50 μl lysate was diluted in 450 μl dilution buffer (0.01% SDS, 1.1% Triton X-100, 167 mM NaCl, and 16.7 mM Tris-HCl, pH 8.1) and incubated overnight at 4°C with 5 μg of anti-Elavl1 (11910-1-AP; Proteintech), anti-Zfp36 (12737-1-AP; Proteintech), and anti-Ybx1 (20339-1-AP; Proteintech), or control IgG Ab, followed by 2-h incubation with protein A/G beads. Beads were washed sequentially with low-salt (150 mM NaCl), high-salt (500 mM NaCl), and LiCl (0.25 M) buffers, and RNA was eluted in 1% SDS/100 mM NaHCO_3_ with Proteinase K (60°C, 2 h). Purified RNA (TRIzol) was reverse transcribed, and target enrichment was quantified by qPCR using primers for *Ccl2*/*Ccl8*, normalized to input RNA and IgG controls.

### *Tet2* catalytic-dependent regulation of *Ccl2* and *Ccl8* mRNA stability

*Tet2* ^KO^ THP1 cells were generated via CRISPR-Cas9 and validated by western blot, and *Tet2*-catalytic mutant were prepared by employing lentiviral transduction in *Tet2*^KO^ cells with puromycin selection prior to decay assays. THP1 monocytes were cultured in RPMI-1640 supplemented with 10% FBS and differentiated into pro-inflammatory macrophages using 100 ng/ml PMA (48 h), followed by 20 ng/ml IFN-γ^+^ and 100 ng/ml LPS (24 h), with differentiation confirmed (>95% CD11b^+^/CD14^+^) by flow cytometry. For mRNA decay assays, differentiated macrophages were treated with 5 μg/ml actinomycin D, harvested at 0–10 h, and analyzed by RT-qPCR using *Ccl2*/*Ccl8*-specific primers).

### Pull-down assay

To investigate Ybx1, Elavl1, and Zfp36 binding to 5mC/5hmC-modified *Ccl2*/*Ccl8* 3′UTRs, we performed biotin-RNA pull-down assays using synthetic 25-nt RNA probes containing AU-rich sequences (*Ccl2*: 5′-biotin-G(5m)CCUUAAGUAAUGUUAAUUCUUAUUUAAGU-3′; *Ccl8*: 5′-biotin-GAAAAG(5m)CUUAUUUAUUUUCCCCAACCUCCC-3′) with site-specific 5mC, and 5hmC modifications were synthesized. *Tet2*^+/+^, *Tet2*^−/−^ THP1–derived pMDMs lysates (500 μg) were incubated with 2-pmol biotinylated probes for 1 h at 4°C, followed by precipitation with streptavidin beads (Beyotime) and stringent washing. Pull-down assay was performed by incubating 5mC and 5hmC oligos of *Ccl2* and *Ccl8* mRNA with cell lysate from THP1-derived pMDMs. Captured proteins were analyzed by western blot using Abs against Ybx1 (11910-1-AP; Proteintech), Elavl1 (20339-1-AP; Proteintech), and Zfp36 (12737-1-AP; Proteintech).

### RNA sequencing

The next-generation RNA sequencing was used to detect the mRNA expression in liver of *Tet2*^WT^, *Tet2*^WT^-CCl_4_, *Tet2*^ΔMye^, and *Tet2*^ΔMye^-CCl_4_ mice. Total RNA was isolated using the Trizol Reagent (#15596026; Invitrogen Life Technologies), after which the concentration, quality, and integrity were determined using a NanoDrop (Thermo Fisher Scientific). Three micrograms of RNA were used as input material for the RNA sample preparations. Sequencing libraries were generated by using TruSeq RNA Sample Preparation Kit (Illumina). Briefly, poly-T oligo-attached magnetic beads were used to purify mRNA from total RNA. Fragmentation was carried out using divalent cations under elevated temperature in an Illumina proprietary fragmentation buffer. First strand cDNA was synthesized by random oligonucleotides and SuperScript II, and the second strand cDNA synthesis was subsequently performed using DNA Polymerase I and RNase H. Remaining overhangs were converted into blunt ends through exonuclease/polymerase activities, and the enzymes were removed. Illumina PE adapter oligonucleotides were ligated to prepare for hybridization after adenylation of the 3′ ends of the DNA fragments. The AMPure XP system (Beckman Coulter) was applied to purify cDNA fragments of the preferred 200-bp DNA fragments with ligated adapter molecules on both ends, which were selectively enriched using Illumina PCR Primer Cocktail in a 15-cycle PCR reaction. Products were purified (AMPure XP system) and quantified using the Agilent high sensitivity DNA assay on a Bioanalyzer 2100 system (Agilent). The sequencing library was then sequenced on a NovaSeq platform (Illumina) by Shanghai Personal Biotechnology Cp. Ltd. The DEGs were analyzed and presented by volcano plot, heat map, GO, GSEA, and KEGG analysis. The Sequence Read Archive (SRA) of the NCBI under accession number is PRJNA1314430.

### WES sequencing and data analysis

WES libraries were prepared from 200 ng of FFPE-derived DNA and 100 ng of matched white blood cell (WBC) gDNA using the VAHTS Universal Plus DNA Library Prep Kit for Illumina V2 (ND627; Vazyme) according to the manufacturer’s protocol, with enzymatic fragmentation performed prior to library construction. Quality-filtered reads were aligned to the hg19 human reference genome using BWA-MEM (v0.7.17), followed by duplicate marking with Picard (v2.20.2) and local realignment around indels using GATK (v4.1.3.0), with sequencing metrics assessed by Bamdst (v1.0.9). Somatic variants were called using VarDict (v1.8.2) with a minimum allele frequency threshold of 2%, and participants were categorized as no CHIP (VAF < 2%), any CHIP (VAF ≥ 2%), or high-confidence CHIP (VAF ≥ 10%) based on variant allele frequencies. The sequencing data are deposited in Sequence Read Archive (SRA) of the NCBI under accession number of PRJNA1375468.

### Statistical analysis

All data were analyzed by using GraphPad Prism software version 9.0. All experiments contained at least three biological repeats, and the littermates were strictly used as the control of all experimental groups involved in the comparison of WT versus KO groups in this study. Two tailed, paired, or unpaired Student’s *t* test was used to analyze the difference of two groups. One-way ANOVA with Tukey’s multiple comparison test or two-way ANOVA with Sidak’s multiple comparison test was used for comparisons of multiple groups. All values were expressed as mean ± SD; *P < 0.05; **P < 0.01; ***P < 0.001. P values <0.05 were considered as statistically significant.

### Online supplemental material

Fig. S1 shows the makers of liver fibrosis and immune cell infiltration relevant for Figs. 1 and 2. Fig. S2 shows engrafted CD45.2 cells and the pMDMs gating strategy and IF staining relevant for Figs. 2 and 3, and DEGs statistic for RNA sequence data relevant for Fig. 4. Fig. S3 shows Ccl2 and Ccl8 expression profiles in different immune cells relevant for Fig. 4 and liver fibrosis marker change after Bindarit treatment relevant for Fig. 5. Fig. S4 shows Ccr2/3 and RNA-interaction protein expression relevant for Fig. 6/Fig. 7 and the cytokines mediating pro-inflammatory effect of pMDMs relevant for Fig. 8. Fig. S5 shows data for BDL-induced liver fibrosis model relevant for Fig. 9 and IL-1β–NLRP3 signaling pathway in our model relevant for discussion section. Table S1 contains the main reagents and kits. Table S2 contains primers for RT-PCR and genotyping of mice. Table S3 contains the Abs list. Table S4 contains the GSEA reports in Fig. 4 A. Table S5 contains the RNA sequence data relevant for Fig. 4 C. Table S6 contains the 3′UTR sequence relevant for Fig. 7, C and D.

## Supplementary Material

Table S1 contains the main reagents and kits.

Table S2 contains primers for RT-PCR and genotyping of mice.

Table S3 contains the Ab list for western blot, IHC, IF, and flow cytometry.

Table S4 contains the GSEA reports in Fig. 4 A.

Table S5 contains the RNA sequence data relevant for Fig. 4 C.

Table S6 contains the 3′UTR sequence relevant for Fig. 7, C and D.

SourceData F1 is the source file for Fig. 1.

SourceData F5 is the source file for Fig. 5.

SourceData F7 is the source file for Fig. 7.

SourceData F8 is the source file for Fig. 8.

SourceData FS1 is the source file for Fig. S1.

## Data Availability

The data underlying Fig. 4, A–C; and Fig. S2 K are openly available in the SRA of the NCBI under accession number PRJNA1314430. The data underlying Fig. 10 A are openly available in the SRA of the NCBI under accession number PRJNA1375468. The rest of the data are available from the corresponding authors upon reasonable request.
