# Supplementary material for: Tet2 deficiency–induced expansion of monocyte-derived macrophages promotes liver fibrosis
Source: J Exp Med. 2025 Dec 26;223(2):e20251114. doi: 10.1084/jem.20251114 (PMC12755866; doi:10.1084/jem.20251114)
Supplement: Table S1 — contains the main reagents and kits. [file jem_20251114_tables1.docx]

**Table S1.** **Main reagents and kits**

| Main Reagents | Cat. no | Brand |
| --- | --- | --- |
| Bindarit | AF283 | MCE |
| Clodronate liposomes | 40337ES08 | YEASEN |
| Thioglycollate medium | SLBZ9877 | Sigma-aldrich |
| Sodium citrate antigen retrieval solution (50×) | YFH5001 | Yifan biology |
| TRIzol Reagent | 254712 | Life technologies |
| 2,2,2-Tribromoethanol | MKCM8927 | Sigma-aldrich |
| Hanks' Balanced Salt Solution (1×) | 2193009 | Gibco |
| BD pharm Lyse Lysing buffer (10×) | 555899 | BD Pharmingen |
| Smart-ECL Enhanced solution | H30100 | Smart-Lifesciences |
| Dmethyl adipimidate dihydrochloride | MKBH5192V | Sigma-aldrich |
| Bovine Serum Albumin | WXBD5147V | Sigma-aldrich |
| PrimeScript RT reagent with gDNA Eraser | RR047A | Takara |
| RIPA lysis buffer | E-BC-R327 | Elabscience |
| DMEM | D6429 | Sigma-aldrich |
| RPMI-1640 | R8758 | Sigma-aldrich |
| Fetal bovine serum(FBS) | 04-001-1ACS | Biological Industries |
| PBS | SH30256.01B | HyClone |
| Collagenase B | 11088807001 | Roche |
| ELISA kit for IV collagen | 20024 | Ruixin biotechnology |
| ELISA kit for HA | 20067 | Ruixin biotechnology |
| ELISA kit for IL-6 | ARG80199 | Arigobio |
| ELISA kit for CCL8 | RX27820 | Ruixin biotechnology |
| ELISA kit for CCL2 | SRE-E14826 | Scrbio |
| Pronase | P5147 | Sigma |
| Hydrogen peroxide | 10011218 | Sinopharm Group Chemical Reagent Co., Ltd. |
| BSA | A8020 | Solarbio |
| hematoxylin stain | R1004 | Shanghai Ruibaohe Biotechnology Co., Ltd. |
| 10× red blood cell lysing buffer | 555899 | BD Pharmingen |
| 4% paraformaldehyde solution | DF1035 | Reagan Bio |
| PBS buffer | SH30256.01 | Hyclone |
| Trizol | 10296010 | Invitrogen |
| Chloroform | 67-66-3 | south test |
| isopropyl alcohol | 67-63-0 | south test |
| 75% ethanol | 67-14-5 | south test |
| DEPC water | NR0001 | Reagan Bio |
| SYBR green | 4913914001 | Roche |
| Lipofectamine 2000 | 11668030 | Invitrogen |
| 10× electrophoresis solution | PS105 | yase |
| 10×T/BST | PS103 | yase |
| 10× transfer solution | PS101 | yase |
| methanol | 10114118 | Shanghai test |
| 10% PAGE gel kit | PG112 | yase |
| 12.5% PAGE gel kit | PG112 | yase |
| Xylene | 10023418 | Sinopharm |
| anhydrous ethanol | 10009218 | Sinopharm |
| 75% ethanol | 80176965 | Shanghai test |
| 30%H2O2 | 10011208 | Shanghai test |
| Normal goat serum | AR0009 | Boster |
| 5%BSA | AR0004 | Boster |
| Rat IgG Immunohistochemistry Kit | SA1055 | Boster |
| Rabbit IgG Immunohistochemistry Kit | SA1022 | Boster |
| EDTA antigen retrieval solution | YF1052 | Yifan Biology |
| Sodium Citrate Antigen Retrieval Solution | YF1050 | Yifan Biology |
| Hydrochloric acid | RR047Q | Takara |
| DAB staining solution | AR1027 | Boster |
| Hematoxylin staining solution | AR1180-1 | Boster |
| Eosin staining solution | AR1180-2 | Boster |
| Neutral resin | 96949-21-2 | Solebao |
| Carbon tetrachloride (CCl_4_) | 10006464 | Shanghai test |
| Thioglycolate | 70157 | Sigma |
| Anti-fluorescence quencher | 0100-01 | SouthernBiotech |
| Triton X-100 | 9002-93-1 | Sigma |
