# Supplementary material for: Tet2 deficiency–induced expansion of monocyte-derived macrophages promotes liver fibrosis
Source: J Exp Med. 2025 Dec 26;223(2):e20251114. doi: 10.1084/jem.20251114 (PMC12755866; doi:10.1084/jem.20251114)
Supplement: Table S2 — contains primers for RT-PCR and genotyping of mice. [file jem_20251114_tables2.docx]

**Table S2.** **Primers for RT-PCR and genotyping of mice**

| Primers | Sequence (5′-3′) |
| --- | --- |
| Lyz-Cre-P1 | CCCAGAAATGCCAGATTACG |
| Lyz-Cre-P2 | CTTGGGCTGCCAGAATTTCTC |
| Lyz-Cre-P3 | TTACAGTCGGCCAGGCTGAC |
| m*Tet2*-C | AGTTCACCCTTCTCATGTGGATACT |
| m*Tet2*-R1 | CTCTTTACCATACTTGATTGGCTCT |
| m*Tet2*-R2 | ATCACCTTAGGTGACTGCATATGGT |
| Alb-Cre-F1 | TGGCAAACATACGCAAGGG |
| Alb-Cre-R1 | CGGCAAACGGACAGAAGCA |
| *Tet2*^f/f^-C | ACACAGAGAAAAGGGTACGTGAA |
| *Tet2*^f/f^-F | ACTCATTAGTGAAATATGTGAGTG |
| *Tet2*^f/f^-R | CTGCTTAGTTCAATGCCAACC |
| mCcl8-F1 | TCTACGCAGTGCTTCTTTGCC |
| mCcl8-R1 | AAGGGGGATCTTCAGCTTTAGTA |
| mCcl2-F1 | TTAAAAACCTGGATCGGAACCAA |
| mCcl2-R1 | GCATTAGCTTCAGATTTACGGGT |
| mColla1-F1 | ACTGCAACATGGAGACAGGTCAGA |
| mColla1-R1 | ATCGGTCATGCTCTCTCCAAACCA |
| mActa2-F1 | TGAAGATCCTGACTGAGCGT |
| mActa2-R1 | TGATGTCACGGACAATCTCA |
| mPdgfr-F1 | TTCCAGGAGTGATACCAGCTT |
| mPdgfr-R1 | AGGGGGCGTGATGACTAGG |
| mTimp1-F1 | GCAACTCGGACCTGGTCATAA |
| mTimp1-R1 | CGGCCCGTGATGAGAAACT |
| mGapdh-F1 | CTAGACACCATGTGCGACGA |
| mGapdh-R1 | ATAGATGGGCACGTTGTGGG |
| mTet2-F1 | AGAGAAGACAATCGAGAAGTCGG |
| mTet2-R1 | CCTTCCGTACTCCCAAACTCAT |
| mCD206-F1 | CTCTGTTCAGCTATTGGACGC |
| mCD206-R1 | CGGAATTTCTGGGATTCAGCTTC |
| mIL-1β-F1 | GCAACTGTTCCTGAACTCAACT |
| mIL-1β-R1 | ATCTTTTGGGGTCCGTCAACT |
| mIL-6-F1 | TAGTCCTTCCTACCCCAATTTCC |
| mIL-6-R1 | TTGGTCCTTAGCCACTCCTTC |
| mIL-1α-F1 | CGAAGACTACAGTTCTGCCATT |
| mIL-1α-R1 | GACGTTTCAGAGGTTCTCAGAG |
| mTNFα-F1 | CCCTCACACTCAGATCATCTTCT |
| mTNFα-R1 | GCTACGACGTGGGCTACAG |
| mIL-2-F1 | TGAGCAGGATGGAGAATTACAGG |
| mIL-2-R1 | GTCCAAGTTCATCTTCTAGGCAC |
| mCcr2-F1 | ATCCACGGCATACTATCAACATC |
| mCcr2-R1 | CAAGGCTCACCATCATCGTAG |
| mCcr3-F1 | TCAACTTGGCAATTTCTGACCT |
| mCcr3-R1 | CAGCATGGACGATAGCCAGG |
| mElval1-F1 | GGATGACATTGGGAGAACGAAT |
| mElval1-R1 | TGTCCTGCTACTTTATCCCGAA |
| mYbx1-F1 | CAGACCGTAACCATTATAGACGC |
| mYbx1-R1 | ATCCCTCGTTCTTTTCCCCAC |
| mZfp36-F1 | CCACCTCCTCTCGATACAAGA |
| mZfp36-R1 | GCTTGGCGAAGTTCACCCA |
| Tet2-sgRNA-antisense | TATAGGAGTATCTAGTAATT |
| Tet2-SgRNA-sense | TCATGGAGCATGTACTACAA |
