# Supplementary material for: Tet2 deficiency–induced expansion of monocyte-derived macrophages promotes liver fibrosis
Source: J Exp Med. 2025 Dec 26;223(2):e20251114. doi: 10.1084/jem.20251114 (PMC12755866; doi:10.1084/jem.20251114)
Supplement: Table S3 — contains the Ab list for western blot, IHC, IF, and flow cytometry. [file jem_20251114_tables3.docx]

**Table S3.** **Antibody list for western blot, IHC, IF, and flow cytometry**

| Antibodies | Cat No. | Clone | Application in this work | Brand |
| --- | --- | --- | --- | --- |
| Ccl8 polyclonal antibody | 20049-1-AP | - | IHC, IF | Proteintech |
| Ccl2 polyclonal antibody | 26161-1-AP | - | IHC, IF, ELISA | Proteintech |
| Anti-CD45.1 | 84325-2-RR | 240921A2 | WB, IHC, IF-P, IF-Fro, ELISA | Proteintech |
| Anti-alpha smooth muscle Actin | ab124964 | ERP5368 | IHC, WB | Abcam |
| Anti-mouse Tet2 Rabbit mAb | 36449 | D6C7K | WB | CST |
| Anti-mouse Collagen Ⅰ | 131984 | - | IHC, IF, WB | Absin |
| Anti-F4/80 | 29414-1-AP |  | IHC, IF | Proteintech |
| LaminB mouse McAb | 66095-1 | - | WB | Proteintech |
| Anti-mouse YBX1 | 20339-1-AP |  | IP, WB | Proteintech |
| Anti-mouse Elval1 | 11910-1-AP |  | IP, WB | Proteintech |
| Anti-mouse Zfp36 | 12737-1-AP |  | IP, WB | Proteintech |
| Anti-mouse iNOS | 22226-1-AP |  | IF | Proteintech |
| Anti-mouse NLRP3 | 30109-1-AP |  | IHC, IF | Proteintech |
| Anti-mouse CD45.2 | 60287-1-Ig | 4E9B2 | IF | Proteintech |
| Anti-mouse Ly6c | 65296-1-Ig | HK1.4 | IF | Proteintech |
| Anti-F4/80 antibody | ab300421 | EPR26545-166 | IF | Abcam |
| Anti-CD68 antibody | ab53444 | FA-11 | IF | Abcam |
| Anti-CD206 antibody | ab300621 | EPR25215-277 | IF | Abcam |
| Recombinant Ccl8 | P6605-10μg | - | RT-PCR | Beyotime |
| Recombinant Ccl2 | P6573-5μg | - | RT-PCR | Beyotime |
| Anti-mouse CD45.1 BV421 | 562895 | 104 | C-flow | BD Pharmingen |
| Anti-mouse CD45.1-Percep-cy5.5 | 110728 | A20 | C-flow | Biolegend |
| Anti-mouse CD45.2-FITC | 103107 | 30-F11 | C-flow | Biolegend |
| Anti-mouse CD11B-APC | 553312 | M1/70 | C-flow | BD Pharmingen |
| Anti-mouse CD11B-Percep-cy5.5 | 550993 | M1/71 | C-flow | BD Pharmingen |
| Anti-mouse Ly6G-PE | 551461 | 1A8 | C-flow | BD Pharmingen |
| Anti-mouse Ly6C-BV421 | 562727 | AL-21 | C-flow | BD Pharmingen |
| Anti-mouse Ly6C-PE-Cy7 | 560593 | AL-21 | C-flow | BD Pharmingen |
| Anti-mouse F4/80-FITC | 4329362 | BMB | C-flow | Ebioscience |
| Anti-mouse F4/80-Percep-BV421 | 565411 | T45-2342 | C-flow | BD Pharmingen |
| Anti-mouse CD206-PE-Cy7 | 4314987 | MR6F3 | C-flow | Ebioscience |
| Anti-mouse CD86-BV510 | 563077 | GL-1 | C-flow | BD Pharmingen |
| Rat anti-mouse CD16/CD32 | 553142 | 2.4G2 | C-flow | BD Pharmingen |
| Goat anti-mouse IgG secondary antibody | 6229 | - | WB | SAB |
| Goat anti-rabbit IgG secondary antibody | 8715 | - | WB | SAB |
| DAPI | ab104139 | - | IF | Abcam |
| In vivoMAb IL-6 | BE0046 | - | In vivo treatment | BioX cell |
| In vivoMAb Anti-CCR2 | BE0457 |  | In vivo treatment | BioX cell |
| In vivoMAb Anti-CCR3 | BE0316 |  | In vivo treatment | BioX cell |
| In vivoMAb Anti-IL-1β | BE0246 |  | In vivo treatment | BioX cell |
| Anti-mouse Fc-block | 553141 | - | C-flow | BD |
| CY3-goat anti-rabbit IgG | ab6939 | - | IHC, IF | Abcam |
| FITC-goat anti-rabbit IgG | ab6785 | - | IHC, IF | Abcam |
