# Supplementary figures and images for: Tet2 deficiency–induced expansion of monocyte-derived macrophages promotes liver fibrosis
Source: J Exp Med. 2025 Dec 26;223(2):e20251114. doi: 10.1084/jem.20251114 (PMC12755866; doi:10.1084/jem.20251114)

Figure 1G

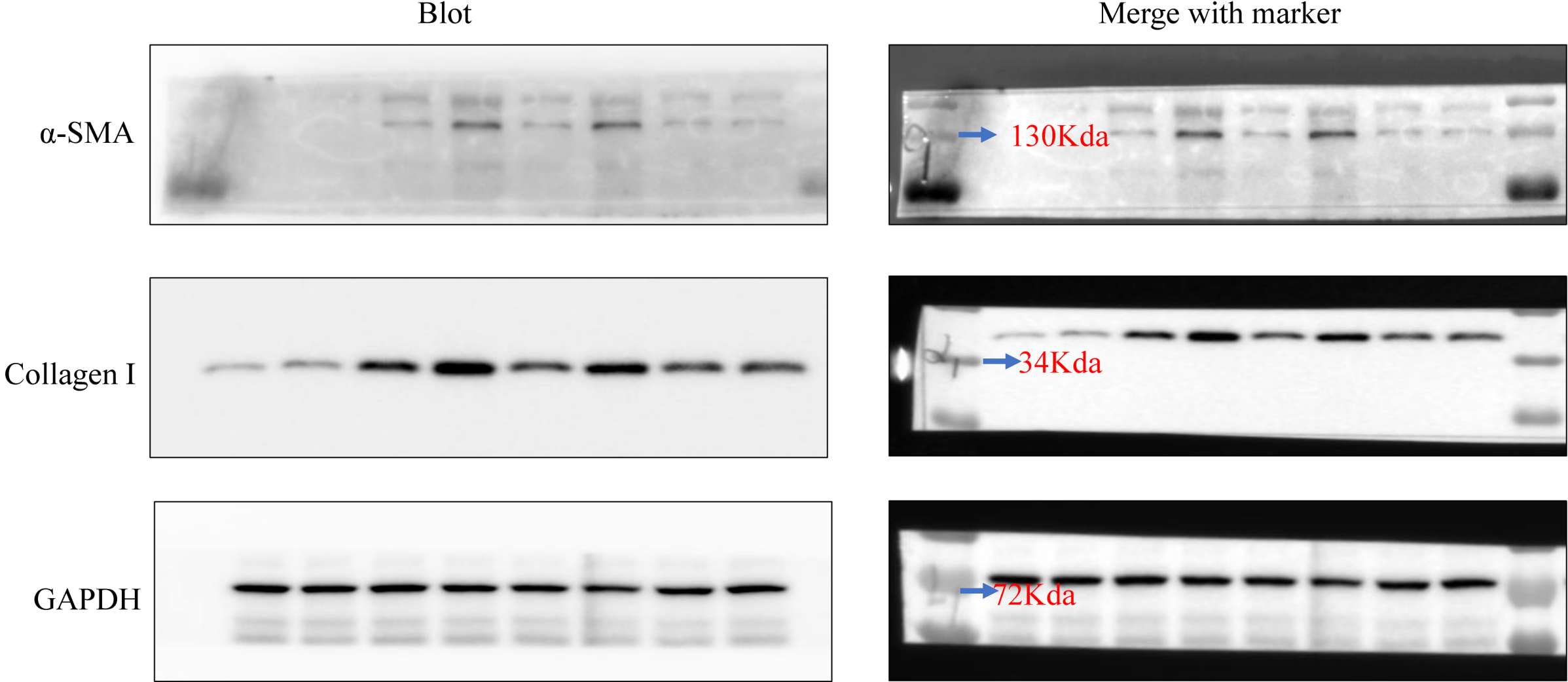

Supplement: SourceData F1 — is the source file for Fig. 1. [file jem_20251114_sourcedataf1.pdf]

Figure 7C

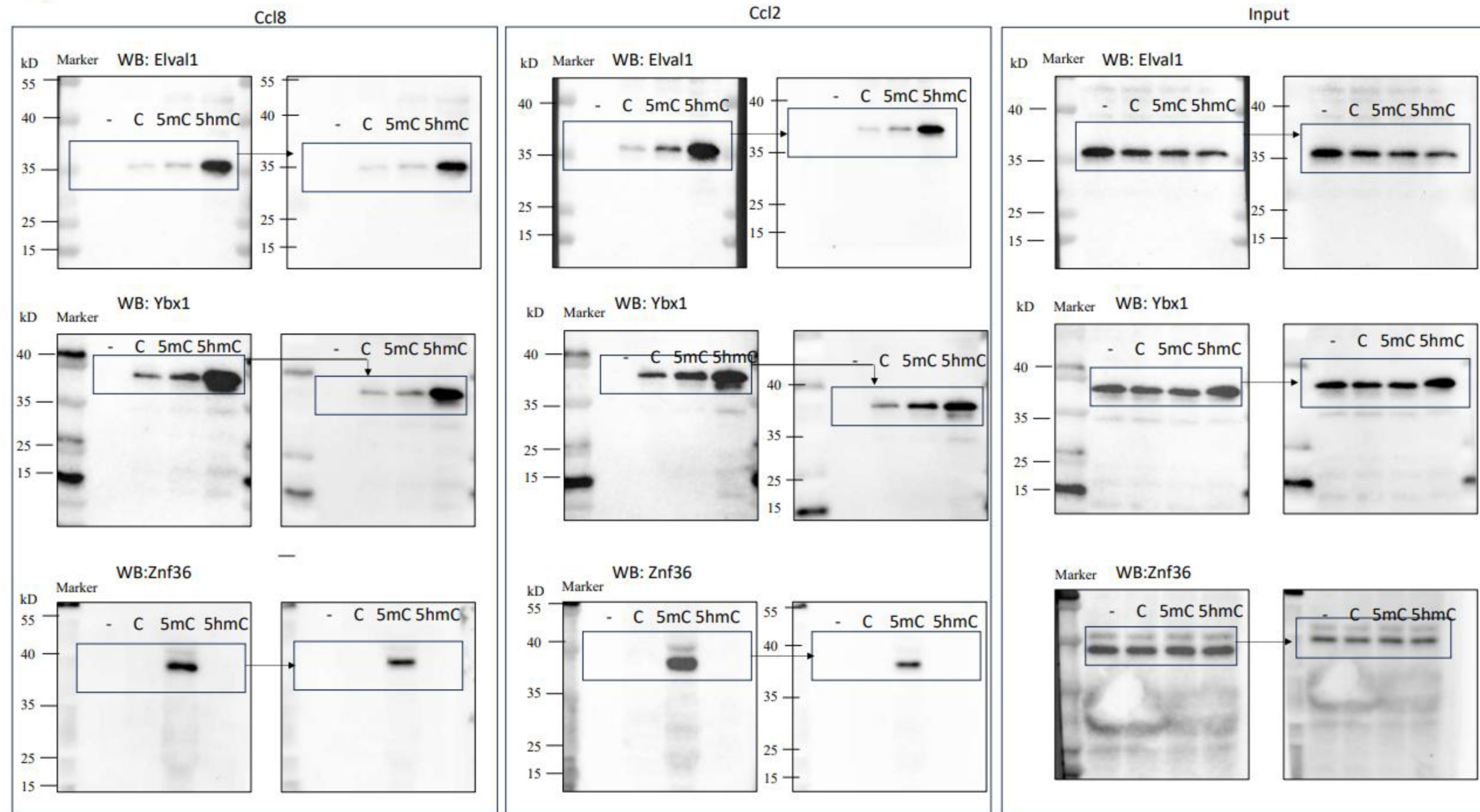

Supplement: SourceData F7 — is the source file for Fig. 7. [file jem_20251114_sourcedataf7.pdf]

Figure 8G

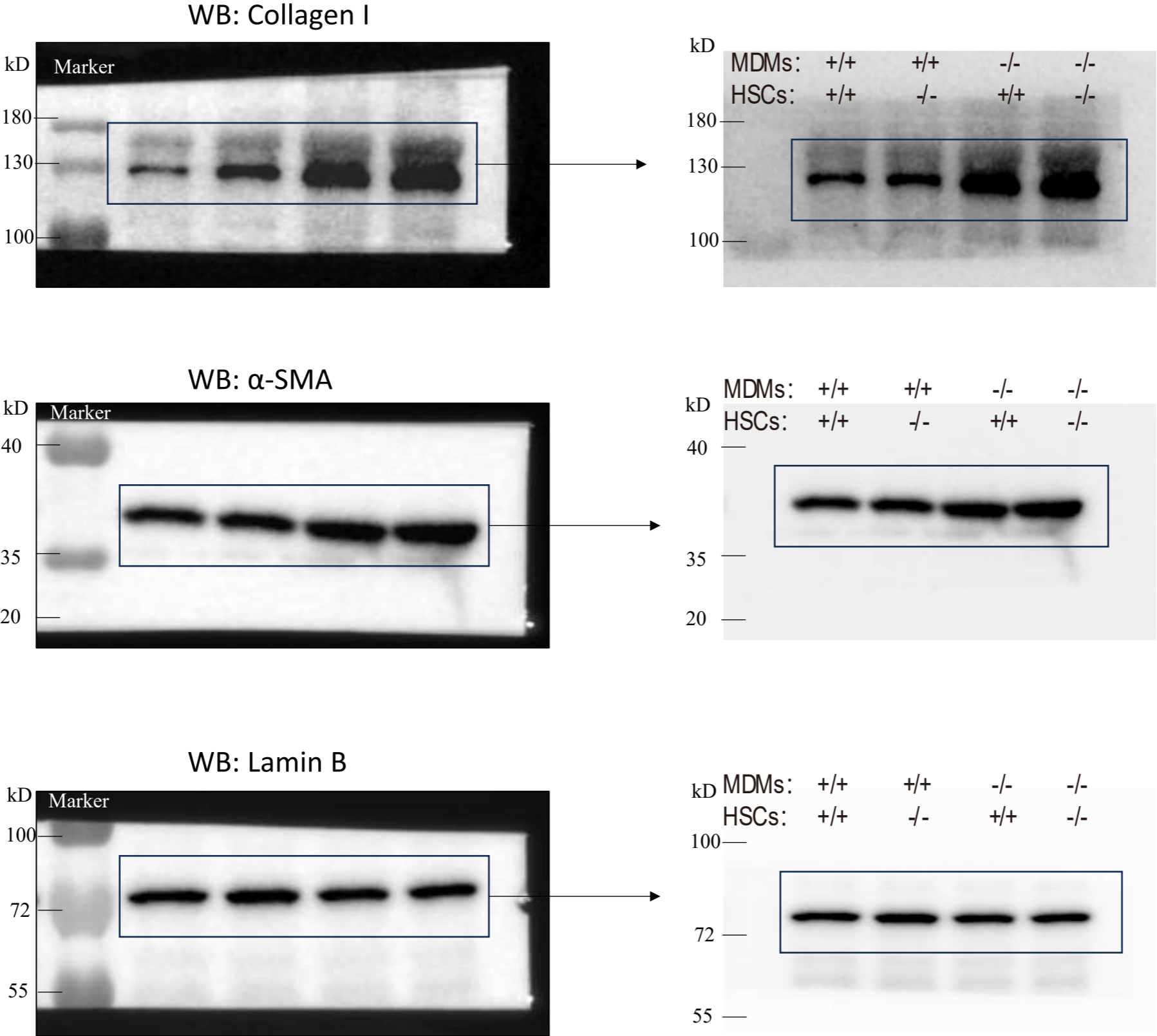

Supplement: SourceData F8 — is the source file for Fig. 8. [file jem_20251114_sourcedataf8.pdf]

Figure S1L

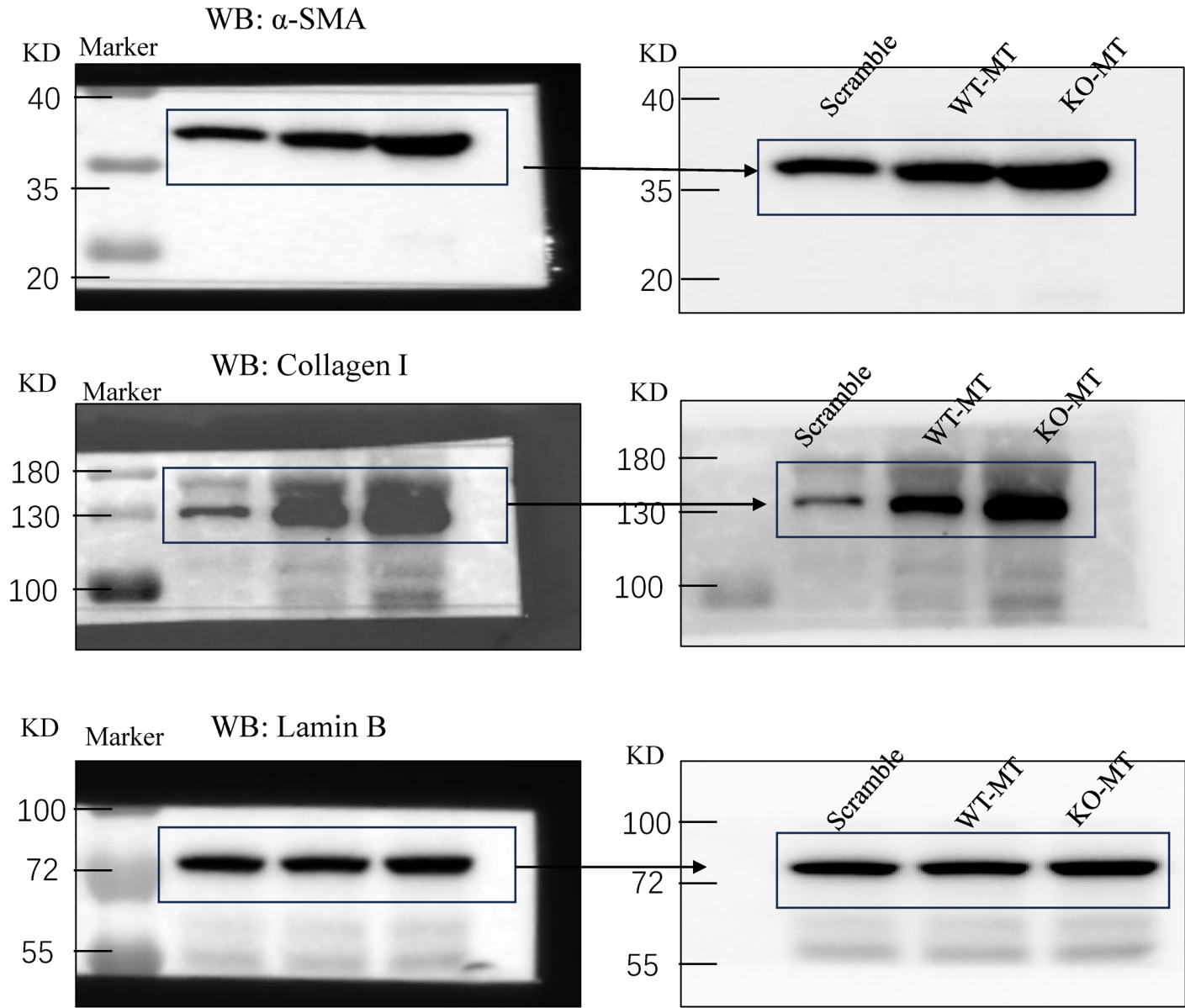

Supplement: SourceData FS1 — is the source file for Fig. S1. [file jem_20251114_sourcedatafs1.pdf]
