# Supplementary material for: Tet2 deficiency–induced expansion of monocyte-derived macrophages promotes liver fibrosis
Source: J Exp Med. 2025 Dec 26;223(2):e20251114. doi: 10.1084/jem.20251114 (PMC12755866; doi:10.1084/jem.20251114)
Supplement: SourceData F5 — is the source file for Fig. 5. [file jem_20251114_sourcedataf5.pdf]

Figure 5H

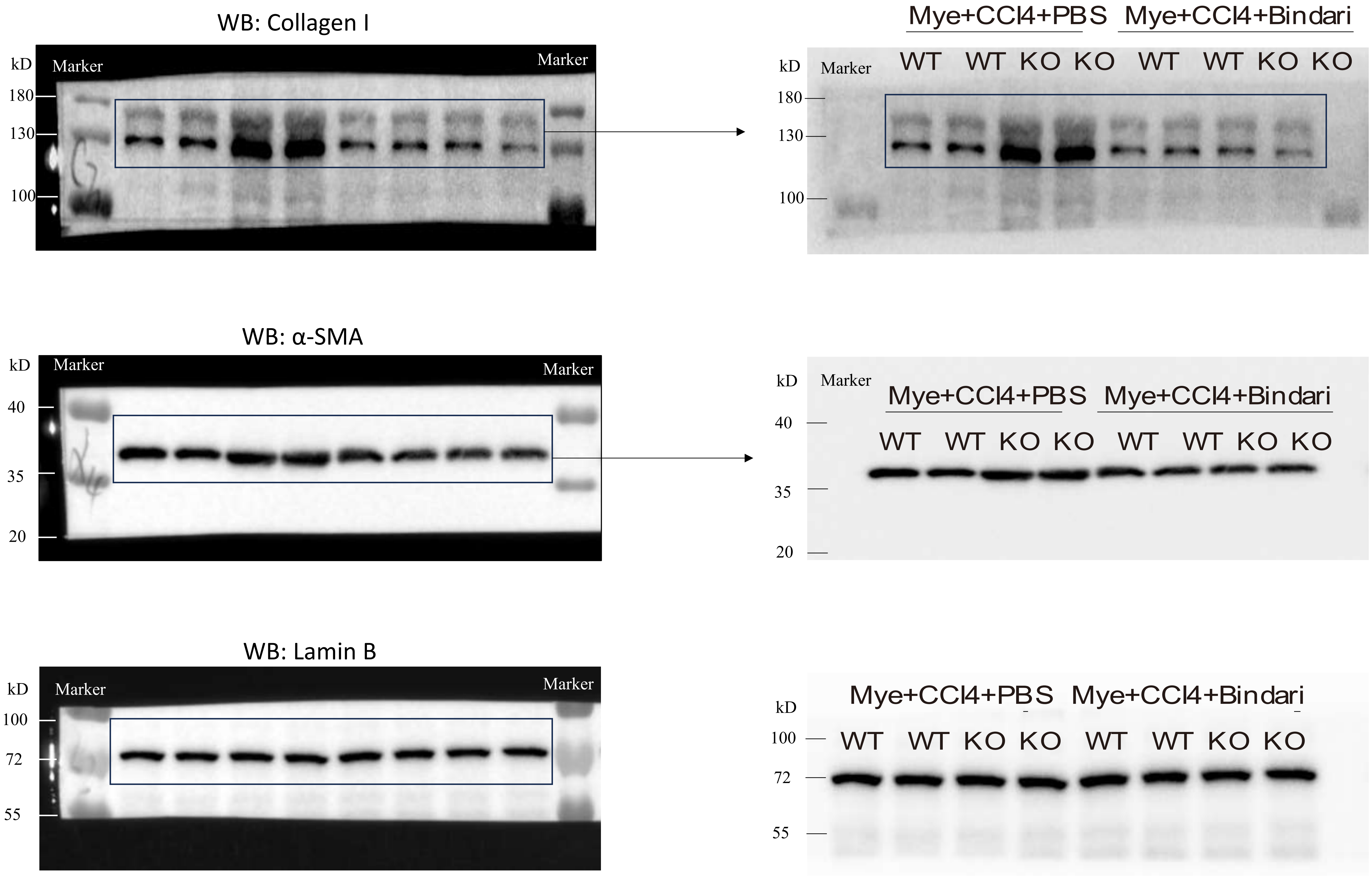

WB: Collagen I
kD
Marker
Marker
180
130
100
Mye+CCl4+PBS
Mye+CCl4+Bindari
kD
Marker
WT
WT
KO
KO
WT
WT
KO
KO
180
130
100
WB: α-SMA
kD
Marker
Marker
40
35
20
kD
Marker
Mye+CCl4+PBS
Mye+CCl4+Bindari
40
WT
WT
KO
KO
WT
WT
KO
KO
35
20
WB: Lamin B
kD
Marker
Marker
100
72
55
Mye+CCl4+PBS
Mye+CCl4+Bindari
kD
100
WT
WT
KO
KO
WT
WT
KO
KO
72
55
